# Investigation of deformation behavior and strain-induced precipitations in Al–Zn–Mg–Cu alloys across a wide temperature range

**DOI:** 10.1038/s41598-024-65669-y

**Published:** 2024-06-26

**Authors:** Qingdong Zhang, Jinrong Zuo, Chen Yang, Yingxiang Xia, Xuedao Shu, Bizhou Mei, Ying Wang, Long Cui

**Affiliations:** 1 Faculty of Mechanical Engineering and Mechanics, Ningbo University, Ningbo, 315211 China; 2 Zhejiang Provincial Key Laboratory of Part Rolling Technology, Ningbo, 315211 China; 3 Ningbo Engineering Research Center for Intelligent Die Casting of New Energy Vehicle Parts, Ningbo, 315211 China; 4 Zhejiang Yiduan Precision Machinery Co., Ltd, Ningbo, 315100 China; 5 Ningbo Junling Mold Technology Co., Ltd, Ningbo, 315800 China

**Keywords:** Al–Zn–Mg–Cu alloy, Arrhenius constitutive model, Hot deformation, Strain-induced precipitation, Microstructure, Materials science, Structural materials, Metals and alloys

## Abstract

This study explores the hot deformation behavior of Al–Zn–Mg–Cu alloy through uniaxial hot compression (200 °C–450°C) using the Gleeble-1500. True stress–strain curves were corrected, and three models were established: the Arrhenius model, strain compensated (SC) Arrhenius model, and strain compensated recrystallization temperature (RT) segmentation-based (TS-SC) Arrhenius model. Comparative analysis revealed the limited predictive accuracy of the SC Arrhenius model, with a 25.12% average absolute relative error (AARE), while the TS-SC Arrhenius model exhibited a significantly improved to 9.901% AARE. Material parameter calculations displayed variations across the temperature range. The SC Arrhenius model, utilizing an average slope method for parameter computation, failed to consider temperature-induced disparities, limiting its predictive capability. Hot processing map, utilizing the Murty improved Dynamic Materials Model (DMM), indicated optimal conditions for stable forming of the Al–Zn–Mg–Cu alloy. Microstructural analysis revealed MgZn_2_ precipitation induced by hot deformation, with crystallographic defects enhancing nucleation rates and precipitate refinement.

## Introduction

Al–Zn–Mg–Cu alloy, renowned for its heat-treatable properties, finds extensive application in aerospace due to its high strength, hot deformation capabilities, and resistance to fatigue^1–3^. Current research on this alloy primarily centers on corrosion resistance^4^, spray forming^5,6^, heat treatment techniques^7,8^, and the microstructure evolution during hot deformation^9,10^. However, there's a noticeable gap in the investigation of its hot deformation behavior across a broad temperature range. Constitutive analysis stands as a well-established method for comprehending the hot deformation behavior of metals^11^. Zhang et al.^12^ investigated the hot deformation behavior of cast Al–Zn–Mg–Cu alloy at temperatures ranging from 300 °C to 450 °C and successfully established the strain-compensated Arrhenius model. Yu et al.^13^ expanded the temperature range of the same material from 250 °C to 450 °C. However, accurately predicting the hot deformation behavior of cast Al–Zn–Mg–Cu alloy across this wide range remains challenging. The established modified Zerilli-Armstrong (MZA) model exhibits a prediction AARE of 12.74%, indicating its inaccuracy. Jiang et al.^14^ studied hot deformation of Ti6Al4V alloy at 600 °C–900 °C, finding negative strain rates at low temperatures. This hindered parameter calculation for the Arrhenius model. Consequently, the constitutive model was only established at higher temperatures.

Additionally, intelligent optimization methods and neural networks have been employed to probe material hot deformation behavior. Chen et al.^15^ used a neural network developed by a genetic algorithm to accurately predict the hot deformation behavior of AA5005 aluminum alloy. While high-accuracy forecasting of neural networks is ensured, it is often considered that the mechanisms of material deformation are ignored^16^. The linear fitting and average slope used by the Arrhenius model to calculate material parameters are the reasons why the Arrhenius model cannot be predicted with extreme precision^15,17^. However, the Arrhenius model is closely related to the thermal activation energy *Q*, which is an important material constant for studying the softening mechanism.

Aluminum alloys with poor room-temperature forming properties are commonly used in hot forming, but their microstructure evolution and hot deformation behaviors are more complicated and directly connected to hot process parameters^18,19^. While the hot processing map is a useful tool to determine the hot process parameters based on the true stress–strain data^18,20^. For example, Wang et al.^21^ established hot processing map of 5A06 aluminum alloy and found that the optimum hot working range of 5A06 aluminum alloy was 350 °C–427 °C and 0.01 s^−1^–1 s^−1^. Li et al^22^ established hot processing map of 7A65 aluminum alloy and analyzed the optimum hot working range as 360 °C–440 °C and 0.1 s^−1^–0.5 s^−1^ and the microstructure evolution of 7A65 aluminum alloy during hot compression was explained by EBSD (Electron back scatter diffraction). Wu et al.^23^ established 3D hot processing map of 6063 aluminum alloy and described the microstructure characteristics of the high efficiency zone as well as the instability zone by optical microscope (OM) as well as TEM (Transmission electron microscope). Nonetheless, no systematic investigation of the hot processing map of Al–Zn–Mg–Cu alloy has been reported, and the dynamic softening behavior during hot processing is still unclear.

This study aims to investigate the hot deformation behavior of Al–Zn–Mg–Cu alloy across a broad temperature range via compression tests. Various Arrhenius models were employed to describe rheological behavior, with the TS-SC Arrhenius model showing promise for accurately predicting deformation over a wide temperature range. Additionally, hot processing map based on Murty improved DMM were utilized to optimize processing windows. EBSD analysis was conducted to characterize microstructures in high-power dissipation and instability zones.

## Materials and methods

**Figure 1:**
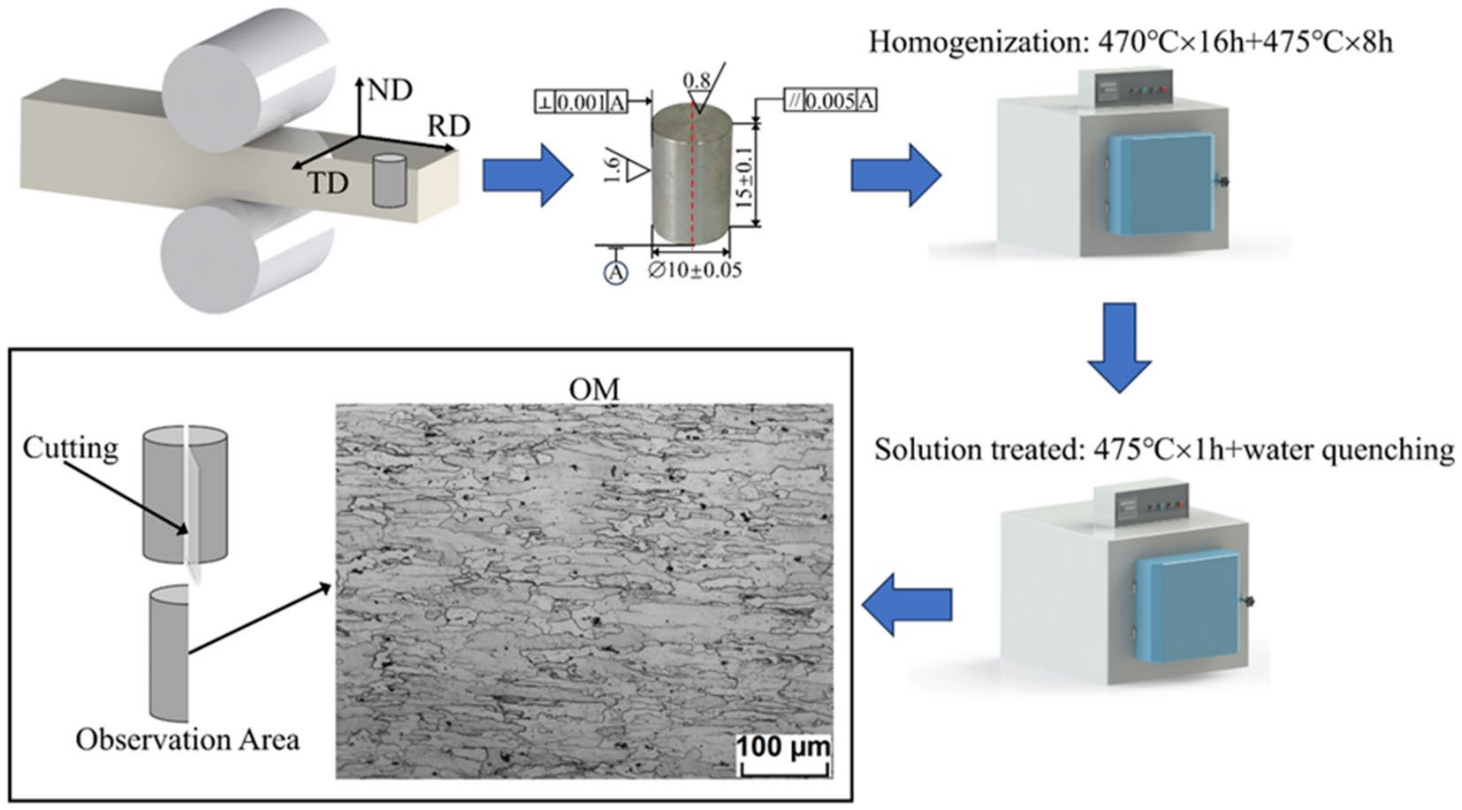
Thermal compression preparation process.

The compressive samples, measuring ϕ 10 mm × 15 mm, were cut along the normal direction (ND) of commercial Al–Zn–Mg–Cu alloy thick plate (supplied by Southwest Aluminum Corporation of China in hot rolled state) using wire electro discharge machining (WEDM). The homogenized operation was conducted at 470 °C for 16 h and 475 °C for 8 h^24^. Following that, the solution treatment was carried out for 1 h at 475 °C. The chemical composition of the samples is shown in Table 1, and the initial microstructure is shown in Fig. 1.

**Table 1:** The chemical composition of Al–Zn–Mg–Cu alloy (wt.%).

Zn	Mg	Cu	Zr	Fe	Si	Ti	Al
8.38	2.07	2.31	0.13	0.092	0.056	0.16	Balance

**Figure 2:**
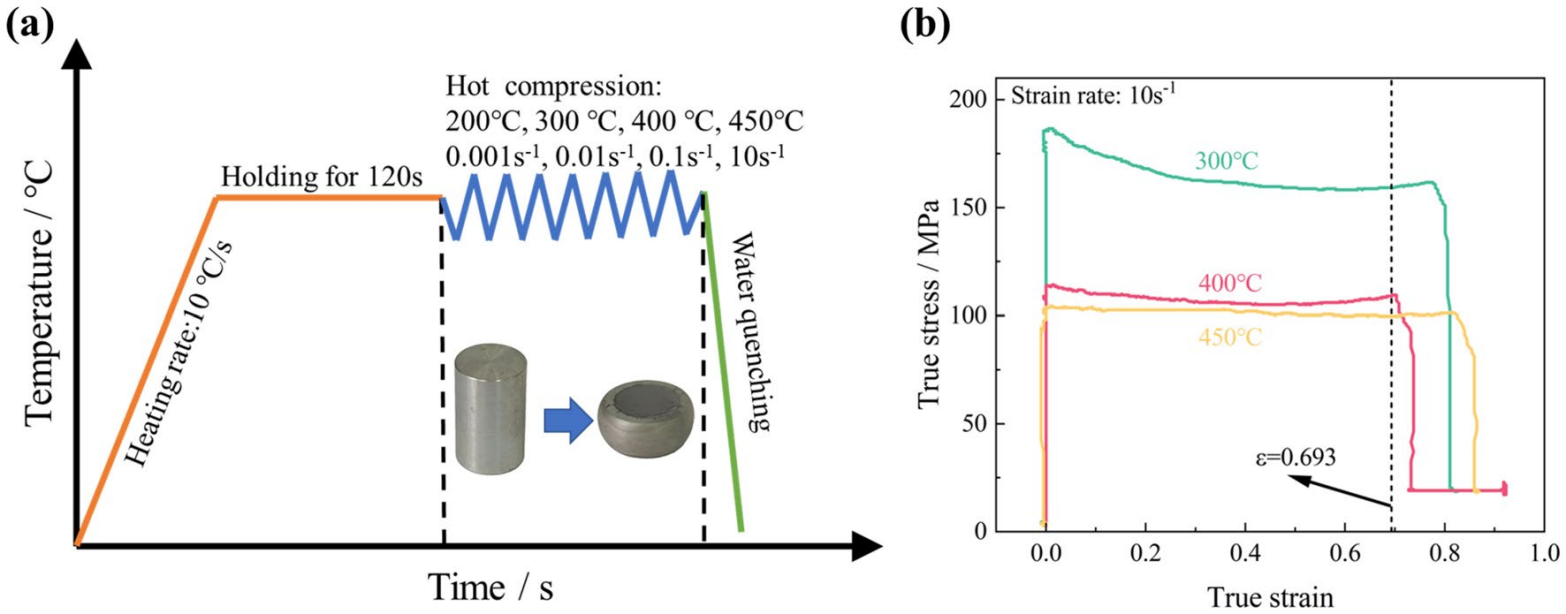
Uniaxial hot compression tests: (**a**) Schematic diagram of the experimental setup; (**b**) Typical true stress–true strain curves.

Before the tests, thermocouples were welded onto the cylindrical samples' surfaces to help keep an isothermal environment^25^. Graphite lubricant was applied to reduce friction between the punches and samples. Uniaxial hot compression tests were conducted on the Gleeble-1500 thermal simulator. The test temperature ranged from 200 °C to 450 °C (the experimental temperatures were 200 °C, 300 °C, 400 °C and 450 °C), while the strain rate varied from 0.001 s^−1^ to 10 s^−1^ (the experimental strain rates were 0.001 s^−1^, 0.01 s^−1^, 0.1 s^−1^ and 10 s^−1^). All samples were heated conductively to the target temperature and held for 120 s to eliminate temperature gradients, with a heating rate of 10 °C/s (Fig. 2a). The deformation set in the hot compression test was 60%, corresponding to a maximum true strain of 0.916. However, maintaining the true strain value of 0.916 at high strain rates during the tests proved challenging for Al–Zn–Mg–Cu alloy (Fig. 2b). To ensure consistency in the modeling range, a true strain value of 0.693 is adopted for the subsequent analysis.

Samples, oriented along the compression direction, were sanded cross-sectionally using 240 to 2000 grit sandpaper and subsequently polished. After mechanical polishing, the strain layer appeared on the samples' surface. Electrolytic polishing (ECP) was used to remove the strain layer from the samples’ surfaces, ensuring the clarity of the Kikuchi patterns and thereby enhancing the reliability of EBSD test results. The electrolyte was composed of 6% HClO_4_ and 94% alcohol, with a control voltage of 30 V applied for 10 s. The electrolyte temperature was maintained at around -10 °C using liquid nitrogen to prevent overheating. Using a Gemini SEM 360 electron microscope, the samples were positioned on an inclined stage at 70° and scanned with a voltage of 20 kV in 2 µm steps, covering an area of approximately 0.047 mm^2^. The obtained data were then analyzed and processed using AZtecCrystal software. Low angle grain boundaries (LAGBs: grain boundaries with misorientations 2° < *θ* < 10°) and high angle grain boundaries (HAGBs: grain boundaries with misorientations *θ* > 10°) were shown as white/green and black lines in the EBSD maps respectively. The twin-jet electropolishing apparatus sample preparation using electrolyte comprising a volumetric ratio of 30% nitric acid to 70% methanol was carried out for TEM analysis, where the working current of TEM is 50–70 mA, and the temperature is -30 °C to -20 °C. The size and area fraction of the second phase were meticulously analyzed using "Image J" software applied to TEM images. To mitigate potential errors, an average value was calculated from three randomly selected TEM images, ensuring a reliable and representative analysis. Phase analysis of the Al–Zn–Mg–Cu alloy was conducted by Rigaku Smartlab SE X-ray diffractometer (XRD) with a scanning speed of 10 °/min.

## Results and discussion

### Correction of friction and temperature-related fluctuations

#### Correction of friction

During hot compression, the sample-indenter contact area expands, boosting friction and causing uneven deformation, termed the 'drum belly' effect. This compromises stress data accuracy, mandating friction correction via experimental curves. The friction correction equation (Eq. (1)) proposed by Ebrahimin and Najafizadeh^26^ is employed to address the impact of friction factors on true stress–strain data.

1 $$\upsigma _{f} = \frac{{\upsigma _{0} c^{2} }}{2(\exp (c) - c - 1)} $$

where σ_*f*_ is the friction-corrected stress, σ_0_ is the experimental stress, and *c* is the friction-correction factor, which can be obtained from Eq. (2):

2 $$ c = \frac{{2mR_{0} \sqrt {h_{0} /h} }}{h} $$

3 $$ m = \frac{(R/h)b}{{(4/\sqrt 3 ) - (2b/3\sqrt 3 )}} $$

The friction coefficient *m* in Eq. (2) can be obtained from Eq. (3), while the other parameters in Eq. (1), (2), (3) can be acquired through Eq. (4), (5), (6):

4 $$ R = R_{0} \sqrt {h_{0} /h} $$

5 $$ b = 4(h\Delta R)/(R\Delta h) $$

6 $$ R_{N} = \sqrt {3\left( {h_{0} /h} \right)R_{0}^{2} - 2R_{M}^{2} } $$

where *h*_0_ and *R*_0_ are the height and radius before hot compression, respectively. *R*_*M*_, *R*_*N*_ and *h* are the radius of the 'drum belly', the radius of the end face and the height of the specimen after hot deformation, respectively. *ΔR* = *R*_*M*_—*R*_*N*_, *Δh* = *h*_*0*_ – *h*.

#### Correction of temperature fluctuations

**Figure 3:**
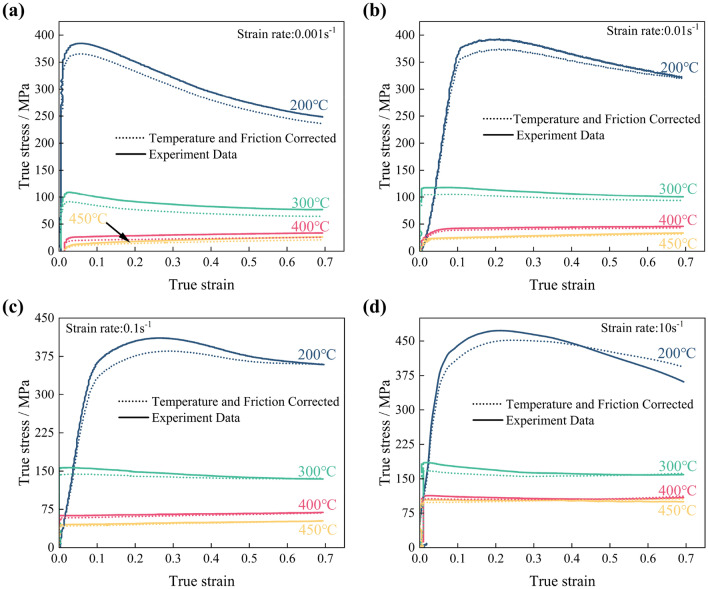
The comparison of true stress–strain between experiment and double corrected curves at different rates: (**a**) 0.001 s^−1^; (**b**) 0.01 s^−1^; (**c**) 0.1 s^−1^; (**d**) 10 s^−1^.

During metal plastic deformation, substantial thermal energy arises. At low strain rates, prolonged deformation facilitates efficient heat exchange. A constant temperature feature in the thermal simulator maintains stable compression temperatures in such cases. However, high strain rates cause rapid energy release, complicating temperature control^27,28^. Goetz and Semiatin^27^ proposed the temperature fluctuation Eq. (7) during isothermal hot compression, combining the previous research:

7 $$\Delta T = \left( {0.95\eta \int {\upsigma } d\varepsilon } \right)/\left( {\rho C_{p} } \right) $$

where *ΔT* is the temperature fluctuation (°C), *η* is the adiabatic correction factor, *σ* is the true stress (MPa), ε is the true strain, *ρ* is the density of the material (g/cm^3^), and *C*_*p*_ is the specific heat of the material (J/ (g⋅K)). The adiabatic correction factor *η* is calculated by Eq. (8)^29,30^:

8 $$ \eta = \left\{ \begin{gathered} 0 \,\,\, \dot{\varepsilon } \le {0}{\text{.001}}s^{ - 1} \hfill \\ {0}{\text{.316}}\log_{10} \left( {\dot{\varepsilon }/\dot{\varepsilon }^{ * } } \right) + {0}{\text{.95\,\,\,0}}{.001}s^{ - 1} { \le } \, {\dot{\varepsilon } < }{1}s^{ - 1} \, \hfill \\ {0}{\text{.95\,\,\,}}\dot{\varepsilon } \ge {1}s^{ - 1} \hfill \\ \end{gathered} \right. $$

where ${\dot{\upvarepsilon}}$ is the strain rate, ${\dot{\upvarepsilon}} ^{ * }$ is the reference strain rate and with a value of 1 s^−1^. Gholamzadeh and Karimi Taheri^31^ proposed Eq. (9) to correct for stress softening caused by temperature fluctuations at high strain rates.

9 $$ \Delta \sigma = \Delta Tf\left. {\frac{{{\text{d}}\sigma }}{{{\text{d}}T}}} \right|_{{\varepsilon ,\dot{\varepsilon }}} $$

where Δσ is the value of stress change and *ΔT* is the value of temperature fluctuations, $(d\upsigma /dT)|_{{{\upvarepsilon  ,\dot{\varepsilon }}}}$ represents the slope of the corrected temperature versus stress under constant strain rate as well as constant strain conditions. Corrections for friction and temperature fluctuations on the true stress–strain curve using the above equation are illustrated in Fig. 3a–d.

Comparing double correction curves with experimental ones shows that friction and temperature fluctuations have distinct impacts on true stress–strain curves. In the elastic deformation stage, friction dominates due to minimal heat generation. With limited deformation and constant contact area, double correction curves closely align with experimental ones. As plastic deformation begins, heat accumulates and friction increases as the deformation area expands^32^. In double correcting experimental curves, heat-induced stress reduction is surpassed by friction-induced stress increases, yielding corrected curves below experimental ones. With increasing strain, accumulated heat leads to temperature fluctuations outweighing friction, causing corrected stress to approach or exceed experimental stress, notably at high strain rates (0.1 s^−1^).

### Arrhenius model

The hot deformation constitutive model primarily describes parameter relationships, such as strain, stress, deformation temperature, and strain rate. The widely applied Arrhenius model focuses on these physical parameters without detailed structural modeling. Through linear regression, material parameters can be derived from experimental data, enabling expression of the constitutive model. The Arrhenius constitutive model, initially proposed by Sellars and McTegart^33^, is represented as follows:

10 $$ {\dot{\upvarepsilon}} = A_{1} \exp (\upbeta \upsigma )\exp ( - Q/RT) \, \upalpha \upsigma  > 1.2 $$

11 $$ {\dot{\upvarepsilon}} = A_{2}\upsigma ^{{n_{1} }} \exp ( - Q/RT) \, {\upalpha  \sigma < }0.8 $$

12 $$ {\dot{\upvarepsilon}} = A_{3} (\sinh \upalpha \upsigma )^{{n_{2} }} \exp ( - Q/RT) \, for \, all \,\upsigma $$

where $\dot{\varepsilon }$ is the strain rate, σ is the stress, *A*_*i*_, *α* and *n*_*i*_ are material parameters,* Q* is the thermal activation energy (kJ/mol), *T* is temperature (K), and R is 8.314. The peak stress Arrhenius model for Al–Zn–Mg–Cu alloy will be established using the linear regression method in this study. Equations (13), (14), (15) can be derived from Eqs. (10), (11), (12).

13 $$ \ln {\dot{\upvarepsilon}} = \upbeta \upsigma  + \ln A_{1} - \frac{Q}{RT} $$

14 $$ \ln {\dot{\upvarepsilon}} = n_{1} \ln\upsigma + \ln A_{2} - \frac{Q}{RT} $$

15 $$ \ln {\dot{\upvarepsilon}} = n_{2} \ln [\sinh (\upalpha \upsigma )] + \ln A_{3} - \frac{Q}{RT} $$

Equations (16), (17), (18) for the derivatives of Eqs. (13), (14), (15), respectively:

16 $$\upbeta (T) = \left[ {\frac{{\partial \ln {\dot{\upvarepsilon}} }}{{\partial\upsigma }}} \right]_{T} $$

17 $$ n_{1} (T) = \left[ {\frac{{\partial \ln {\dot{\upvarepsilon}} }}{{\partial \ln\upsigma }}} \right]_{T} $$

18 $$ n_{2} (T) = \left[ {\frac{{\partial \ln {\dot{\upvarepsilon}} }}{{\partial \ln [\sinh (\upalpha \upsigma )]}}} \right]_{T} $$

**Figure 4:**
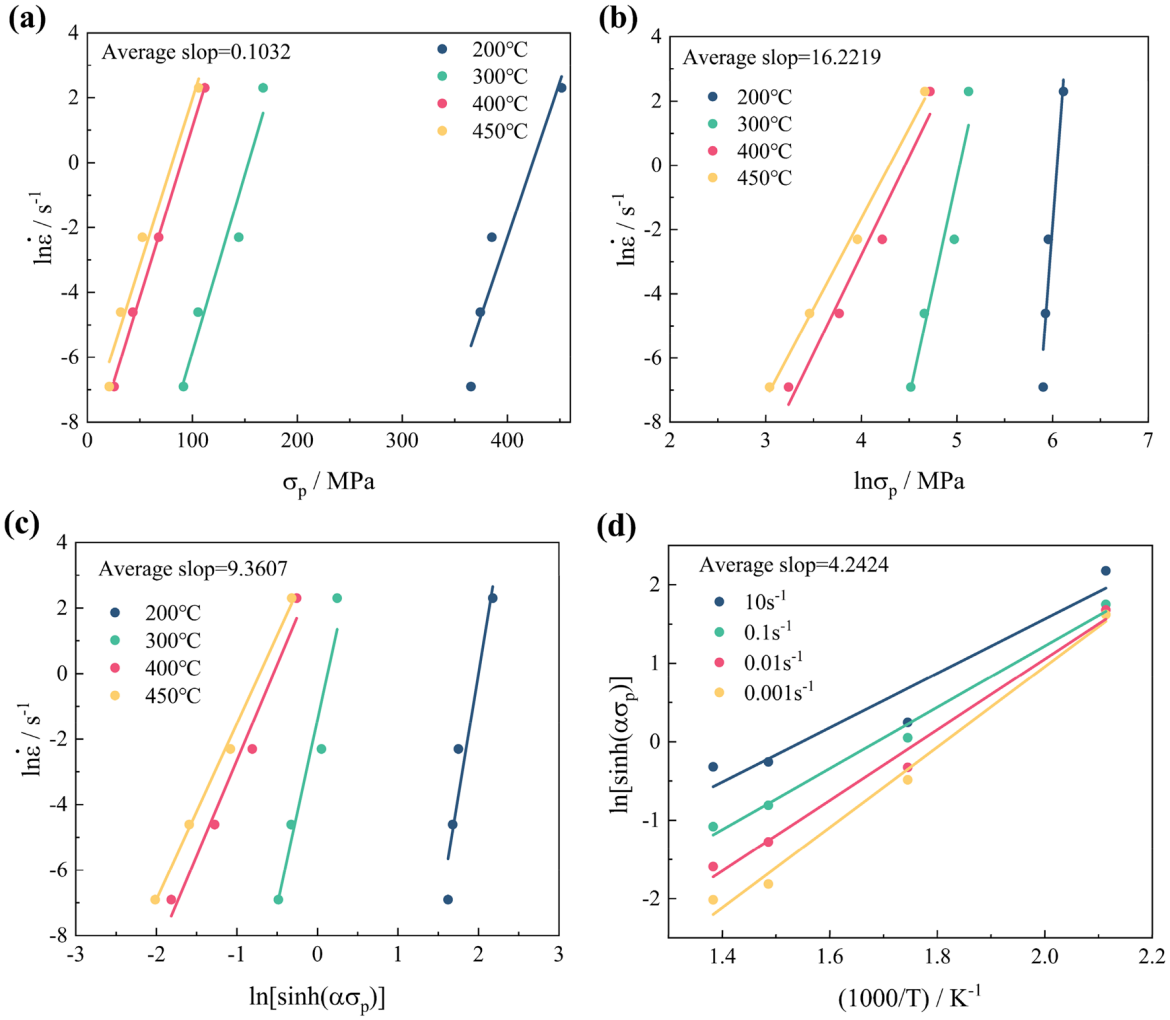
Calculation of material parameters: (**a**) ${\text{ln}}\dot{\varepsilon }{{ - \sigma }}$; (**b**) ${\text{ln}}\dot{\varepsilon }{\text{ - ln}}\sigma$; (**c**) ${\text{ln(}}\dot{\varepsilon }{\text{) - ln[sinh(}}\alpha \sigma {)]}$; (**d**) ${\text{ln[sinh(}}\alpha \sigma {)] - }\left( {1/T} \right)$.

The slopes of the straight lines obtained from Fig. 4a and b correspond to the material constants β and *n*', respectively. The value of α can be calculated using the expression: α = [β/*n*_2_] _T_. Similarly, the slopes of the straight lines acquired from Fig. 4c and d correspond to the material constants *n* and *M*, respectively. Utilizing the results above in conjunction with Eq. (19), the thermal activation energy *Q* can be calculated.

19 $$ Q = R\left\{ {\frac{{\partial \ln \left( {\dot{\varepsilon }} \right)}}{\partial \ln [sinh(\alpha \sigma )]}} \right\}_{T} \left\{ {\frac{\partial \ln [sinh(\alpha \sigma )]}{{\partial \left( {{1 \mathord{\left/ {\vphantom {1 T}} \right. \kern-0pt} T}} \right)}}} \right\}_{{\dot{\varepsilon }}} = RnM $$

By utilizing the linear intercept *h* from Fig. 4c and the expression: ln*A* = *Q*/(*RT*) + *h*, the value of ln*A* is calculated. At this stage, all the material constants have been determined, allowing the establishment of the peak stress Arrhenius model as follows:

20 $$ {\dot{\upvarepsilon}} = 6.8783 \times 10^{25} [\sinh (0.00636\upsigma )]^{9.3607} \exp \left( { - \frac{{{ 330}{\text{.16}}}}{{{\text{R}}T}}} \right) $$

#### SC Arrhenius model

**Figure 5:**
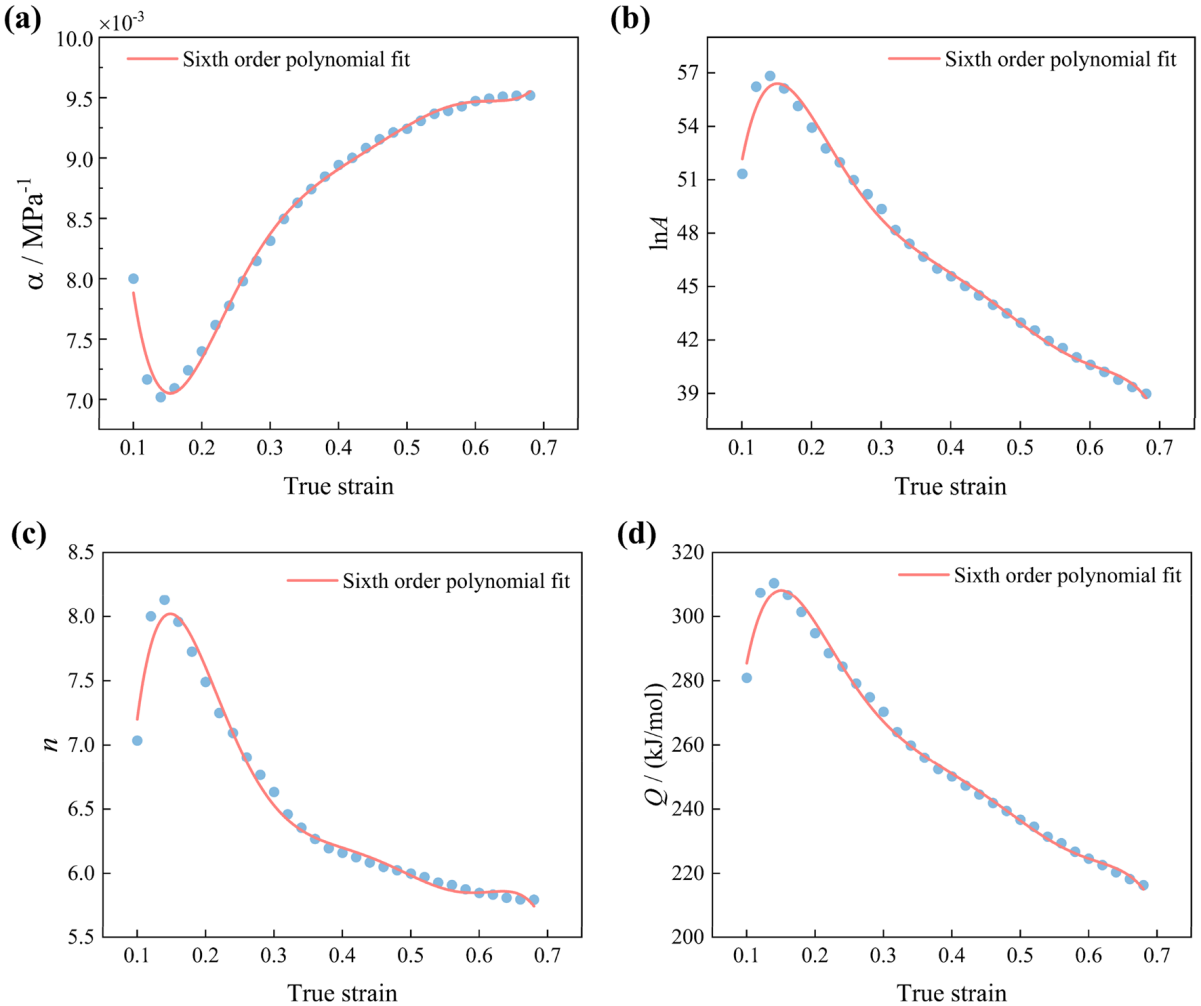
Sixth order polynomial fit of material parameters: (**a**) α; (**b**) ln*A*; (**c**) *n*; (**d**) *Q*.

The Arrhenius model's accuracy in predicting stresses at different strains solely based on material parameters obtained from stress values at a single strain is limited. The SC Arrhenius model (Eq. (21)) is introduced to enhance the prediction accuracy in alloy materials. In this study, the material parameters are calculated for different strains and then fitted to each strain using the polynomial fitting method. Strain compensation points are selected within the range of 0.1 to 0.693, with intervals of 0.02 true strain. Subsequently, a sixth-order polynomial (Eq. (22)) is used to fit the material parameters at each compensation point. The fitted polynomial coefficients are listed in Table 2, and the fitting results are shown in Fig. 5a–d.

21 $$ \left\{ \begin{gathered} Z = {\dot{\upvarepsilon}} \exp \left( {Q/{\text{R}}T} \right) \hfill \\\upsigma = \frac{1}{\upalpha }\ln \left\{ {\left( \frac{Z}{A} \right)^{\frac{1}{n}} + \left[ {\left( \frac{Z}{A} \right)^{\frac{2}{n}} + 1} \right]^{\frac{1}{2}} } \right\} \hfill \\ \end{gathered} \right. $$

where R is the gas constant (the value is 8.314 J/(mol·K)), *T* is the temperature (K), Z is the Zener-Hollomon parameter.

22 $$ \left\{ \begin{gathered} \upalpha \left(\upvarepsilon \right) = B_{0} + B_{1}\upvarepsilon + B_{2}\upvarepsilon ^{2} + B_{3}\upvarepsilon ^{3} { + {\rm B}}_{{4}}\upvarepsilon ^{4} + B_{5}\upvarepsilon ^{5} + B_{6}\upvarepsilon ^{6} \hfill \\ Q\left(\upvarepsilon \right) = C_{0} + C_{1}\upvarepsilon + C_{2}\upvarepsilon ^{2} + C_{3}\upvarepsilon ^{3} { + }C_{{4}}\upvarepsilon ^{4} + C_{5}\upvarepsilon ^{5} + C_{6}\upvarepsilon ^{6} \hfill \\ n\left(\upvarepsilon \right) = D_{0} + D_{1}\upvarepsilon + D_{2}\upvarepsilon ^{2} + D_{3}\upvarepsilon ^{3} { + }D_{{4}}\upvarepsilon ^{4} + D_{5}\upvarepsilon ^{5} + D_{6}\upvarepsilon ^{6} \hfill \\ \ln A\left(\upvarepsilon \right) = E_{0} + E_{1}\upvarepsilon + E_{2}\upvarepsilon ^{2} + E_{3}\upvarepsilon ^{3} { + {\rm E}}_{{4}}\upvarepsilon ^{4} + E_{5}\upvarepsilon ^{5} + E_{6}\upvarepsilon ^{6} \hfill \\ \end{gathered} \right. $$

where *B*_n_, *C*_n_, *D*_n_, and *E*_n_ are the coefficients of the polynomial functions.

**Table 2:** Coefficients of 6th polynomial fitting functions of material constants.

*α*		*Q*		*n*		*ln A*	
*B*_*0*_	0.020246	*C*_*0*_	− 92.6878	*D*_*0*_	− 7.4881	*E*_*0*_	− 18.5607
*B*_*1*_	− 0.25405	*C*_*1*_	7908.1918	*D*_*1*_	310.0602	*E*_*1*_	1479.8655
*B*_*2*_	1.8367	*C*_*2*_	− 58,629.64	*D*_*2*_	− 2324.6747	*E*_*2*_	− 10,980.91
*B*_*3*_	− 6.4065	*C*_*3*_	209,735.5186	*D*_*3*_	8372.1022	*E*_*3*_	39,325.213
*B*_*4*_	11.8878	*C*_*4*_	− 398,407.443	*D*_*4*_	− 15,901.691	*E*_*4*_	− 74,780.35
*B*_*5*_	− 11.2864	*C*_*5*_	385,924.1785	*D*_*5*_	15,352.835	*E*_*5*_	72,495.935
*B*_*6*_	4.3122	*C*_*6*_	− 150,027.439	*D*_*6*_	− 5940.6233	*E*_*6*_	− 28,198.31

**Figure 6:**
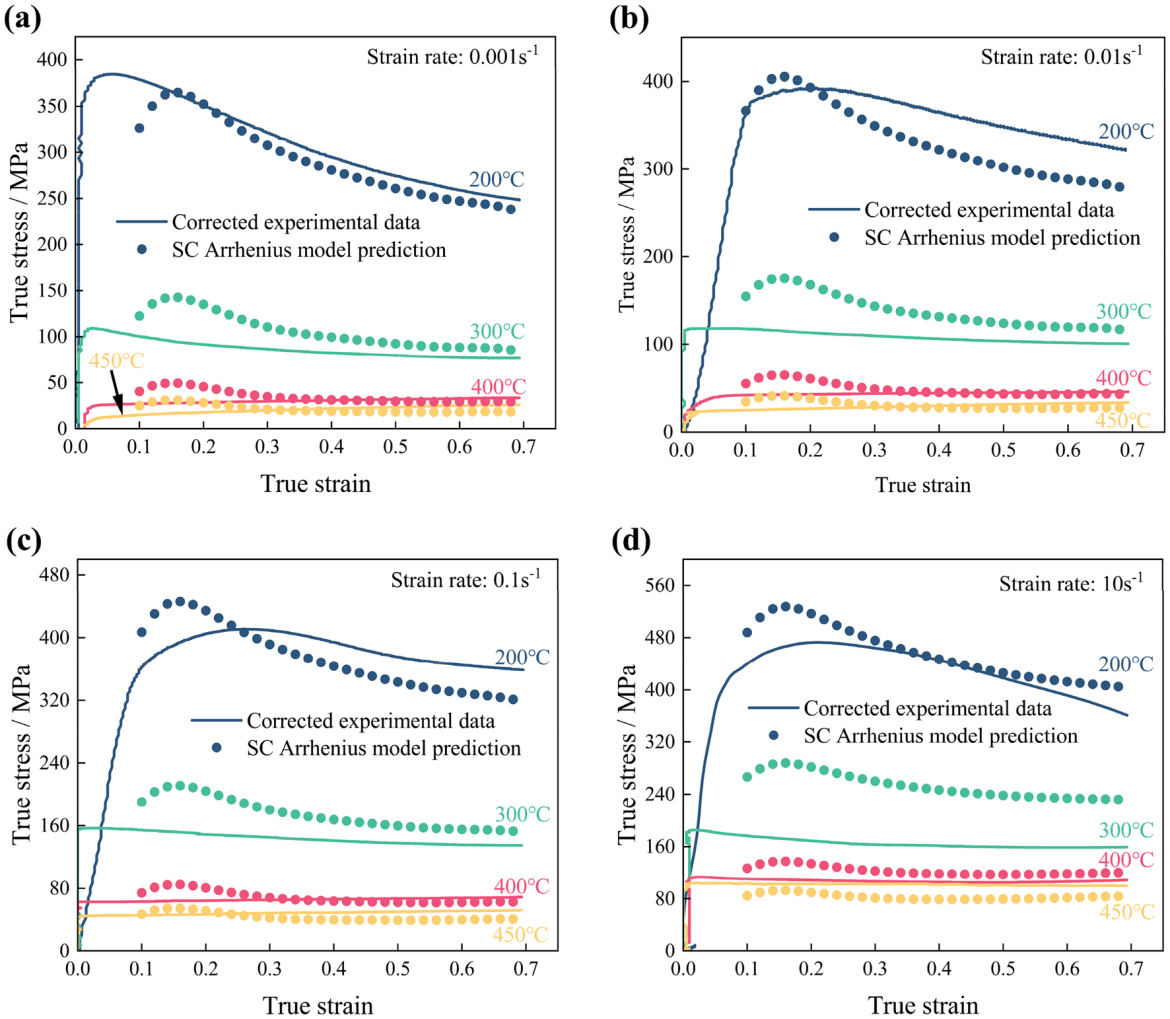
Comparison between double-corrected true stress–strain curves and SC Arrhenius model prediction results at different strain rates: (**a**) 0.001 s^−1^; (**b**) 0.01 s^−1^; (**c**) 0.1 s^−1^; (**d**) 10 s^−1^.

**Figure 7:**
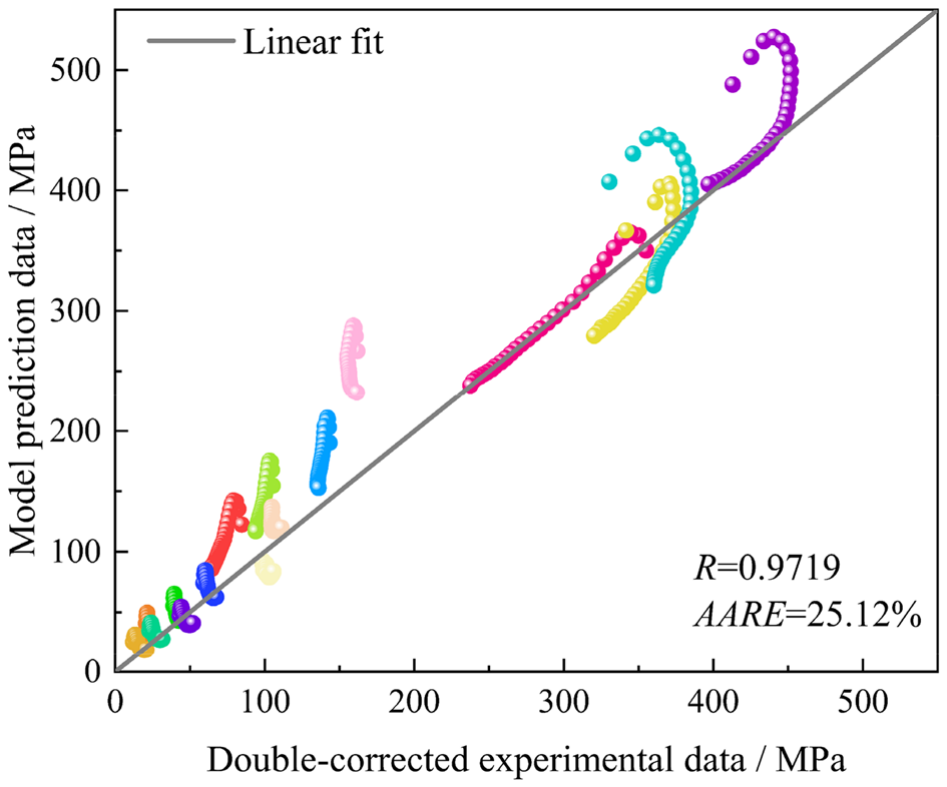
Prediction error of the SC Arrhenius model.

The predicted stresses under all deformation conditions are calculated by the SC Arrhenius model, as illustrated in Fig. 6a–d. Subsequently, the SC Arrhenius model's predictive capacity is assessed by the correlation coefficient *R* (Eq. (23)), and the AARE (Eq. (24)). The prediction error maps are depicted in Fig. 7, where differently colored dots represent predicted stress values under varying deformation conditions. The SC Arrhenius model's error is primarily noticeable in low-temperature, high-strain-rate deformation conditions.

The material parameters of the SC Arrhenius model are computed using an average slope. It's evident from Fig. 4a–d that these slopes substantially vary over a wide temperature range. Using average slopes leads to deviations from the true values of the material parameters at certain deformation temperatures, which challenges the SC Arrhenius model to accurately predict the hot deformation behavior of the Al–Zn–Mg–Cu alloy over such a wide temperature range.

23 $$ R = \frac{{\sum\limits_{i = 1}^{N} {\left( {\upsigma _{E}^{i} - {\overline{\sigma }}_{E} } \right)\left( {\upsigma _{c}^{i} - {\overline{\sigma }}_{c} } \right)} }}{{\sqrt {\sum\limits_{i = 1}^{N} {\left( {\upsigma _{E}^{i} - {\overline{\sigma }}_{E} } \right)^{2} } } \sqrt {\sum\limits_{i = 1}^{N} {\left( {\upsigma _{c}^{i} - {\overline{\sigma }}_{c} } \right)^{2} } } }} $$

24 $$ AARE = \frac{1}{N}\sum\limits_{i = 1}^{N} {\left| {\frac{{\upsigma _{E}^{i} -\upsigma _{c}^{i} }}{{\upsigma _{c}^{i} }}} \right|} { \times }100\% $$

where $\sigma_{E}^{i}$ is the predicted values, $\upsigma _{c}^{i}$ is the tested values, $\overline{\sigma }_{E}$ is the average $\sigma_{E}$, $\overline{\sigma }_{c}$ is the average $\sigma_{c}$, *N* is the number of values.

#### TS-SC Arrhenius constitutive model

**Figure 8:**
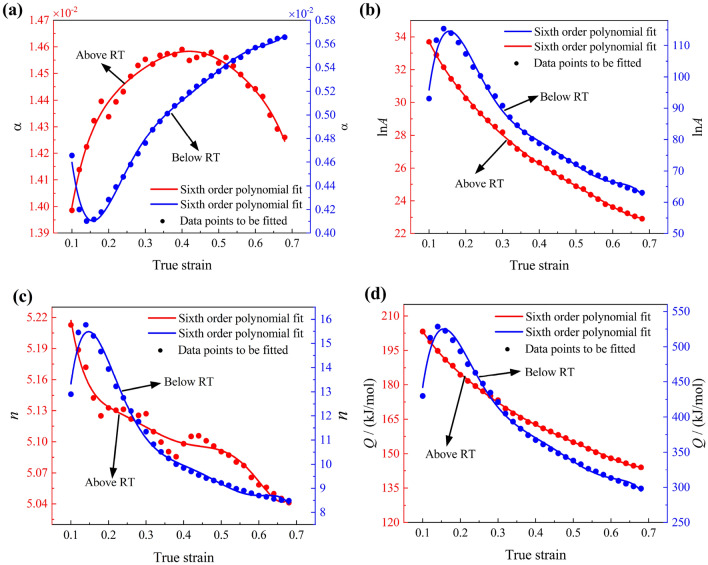
Sixth order polynomial fit of material parameters: (**a**) *α* above (left axis) and below (right axis) the RT; (**b**) ln*A* above (left axis) and below (right axis) the RT; (**c**) *n* above (left axis) and below (right axis) the RT; (**d**) *Q* above (left axis) and below (right axis) the RT.

**Figure 9:**
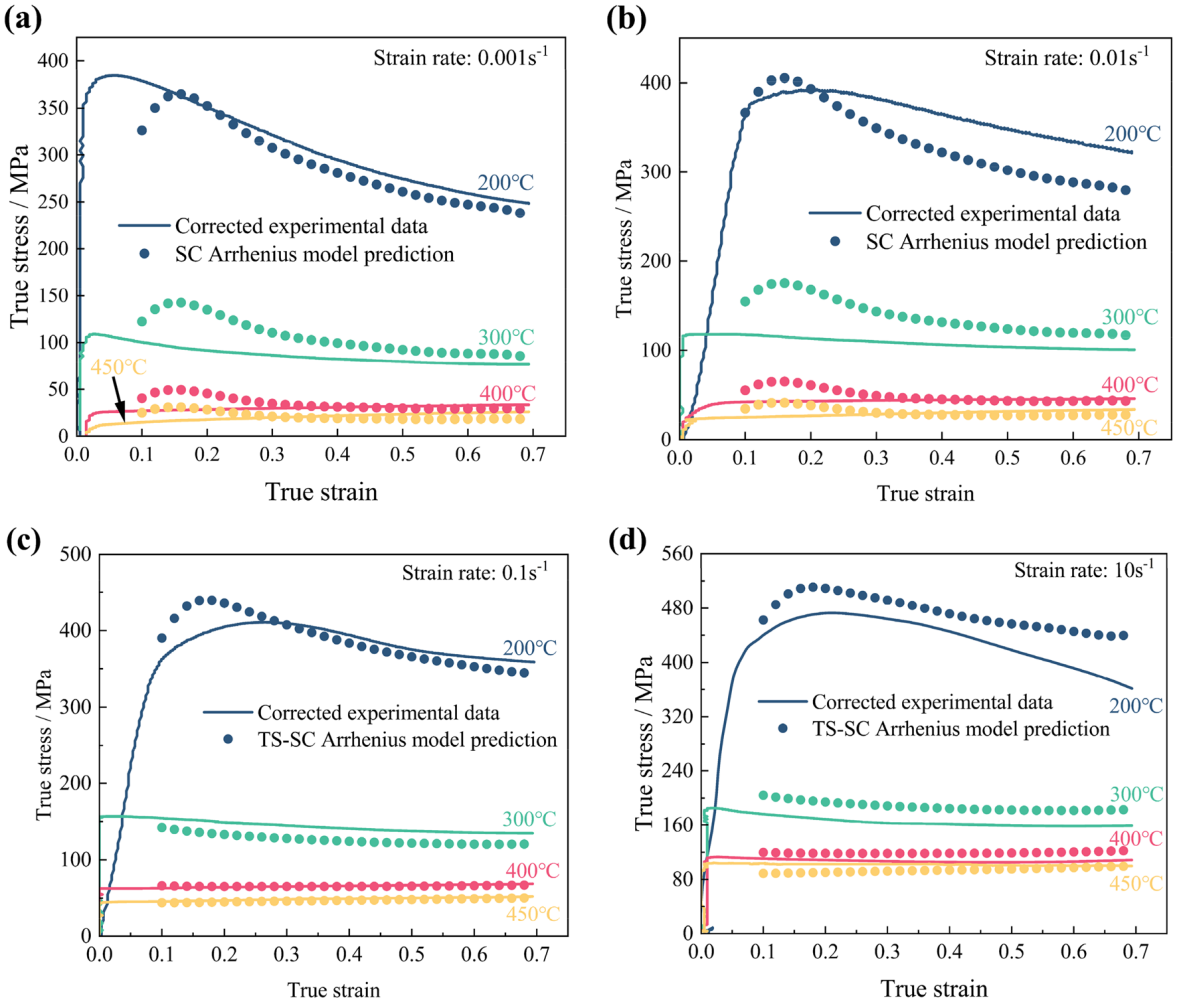
Comparison between double-corrected true stress–strain curves and TS-SC Arrhenius model prediction results at different strain rates: (**a**) 0.001 s^−1^; (**b**) 0.01 s^−1^; (**c**) 0.1 s^−1^; (**d**) 10 s^−1^.

The SC Arrhenius model's material parameters are typically calculated using an average slope, but Fig. 4a–d illustrates significant variations in these slopes across a wide temperature range. This averaging approach results in deviations from true values at certain deformation temperatures, hindering accurate prediction of the Al–Zn–Mg–Cu alloy's hot deformation behavior. To address this, a study based on room temperature (RT) behavior was conducted. RT (approximately 0.4Tm, where Tm is the melting temperature) served as the segmentation point for the constitutive model. Stress–strain data at 300 °C, close to RT, were concurrently utilized to establish a two-stage SC Arrhenius model, ensuring model continuity across temperatures. As shown in Fig. 8a–d, material parameters for both temperature zones were fitted using sixth-order polynomials (Table 3). The significant differences in material parameters between temperature intervals highlight the challenge of accurately describing hot deformation behavior with the SC Arrhenius model's average slope approach. Figure 9a–d depicts stresses calculated using the TS-SC Arrhenius model, with the less inaccurate of the two SC Arrhenius models chosen as the final stress prediction model at 300 °C.

**Table 3:** Coefficients of 6th polynomial fitting functions of material constants.

Deformation type	*α*		*Q*		*n*		*lnA*	
Above RT 300 °C ≤ T ≤ 450 °C	*F*_*0*_	0.012351	*G*_*0*_	243.0499	*H*_*0*_	5.8094	*I*_*0*_	40.799
	*F*_*1*_	0.029644	*G*_*1*_	− 621.201	*H*_*1*_	− 11.9151	*I*_*1*_	− 109.9539
	*F*_*2*_	− 0.18371	*G*_*2*_	3161.493	*H*_*2*_	86.9655	*I*_*2*_	549.3781
	*F*_*3*_	0.62749	*G*_*3*_	− 11,312.12	*H*_*3*_	− 331.9991	*I*_*3*_	− 1961.469
	*F*_*4*_	− 1.186	*G*_*4*_	23,448.874	*H*_*4*_	685.223	*I*_*4*_	4082.7589
	*F*_*5*_	1.156	*G*_*5*_	− 25,278.29	*H*_*5*_	− 722.4631	*I*_*5*_	− 4425.915
	*F*_*6*_	− 0.45632	*G*_*6*_	10,925.64	*H*_*6*_	303.9136	*I*_*6*_	1923.6073
Below RT 200 °C ≤ T < 300 °C	*F’*_*0*_	0.011402	*G’*_*0*_	− 758.6882	*H’*_*0*_	− 26.5613	*I’*_*0*_	− 178.1591
	*F’*_*1*_	− 0.13867	*G’*_*1*_	24,713.485	*H’*_*1*_	843.2509	*I’*_*1*_	5645.2255
	*F’*_*2*_	0.99219	*G’*_*2*_	− 179,601.9	*H’*_*2*_	− 6326.922	*I’*_*2*_	− 41,096.234
	*F’*_*3*_	− 3.4376	*G’*_*3*_	633,274.61	*H’*_*3*_	22,747.131	*I’*_*3*_	145,111.488
	*F’*_*4*_	6.351	*G’*_*4*_	− 1188,175	*H’*_*4*_	− 43,147.49	*I’*_*4*_	− 272,540.58
	*F’*_*5*_	− 6.0062	*G’*_*5*_	1139,380.1	*H’*_*5*_	41,621.713	*I’*_*5*_	261,523.063
	*F’*_*6*_	2.2859	*G’*_*6*_	− 4393,334	*H’*_*6*_	− 16,095.77	*I’*_*6*_	− 100,882.21

**Figure 10:**
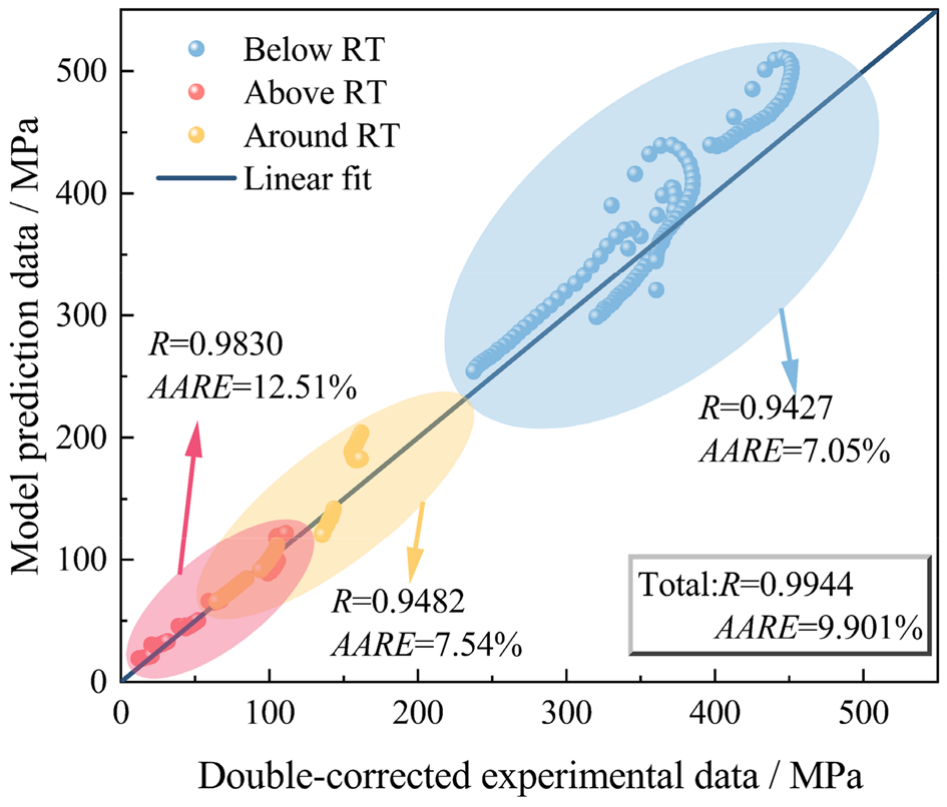
Prediction error of the TS-SC Arrhenius model.

The correlation coefficient *R* as well as the AARE are also used to evaluate the prediction capability of the TS-SC Arrhenius model, as shown in Fig. 10. From the results, the error of the TS-SC Arrhenius model relative to the SC Arrhenius model is reduced from 25.12 to 9.901%, and the prediction capability is improved. In order to have a better understanding of the prediction capability of the TS-SC Arrhenius model, the segments are evaluated. The TS-SC Arrhenius model shown in Fig. 10 has AARE of 12.51% in prediction above the RT, 7.54% around the RT and 7.05% below the RT.

**Figure 11:**
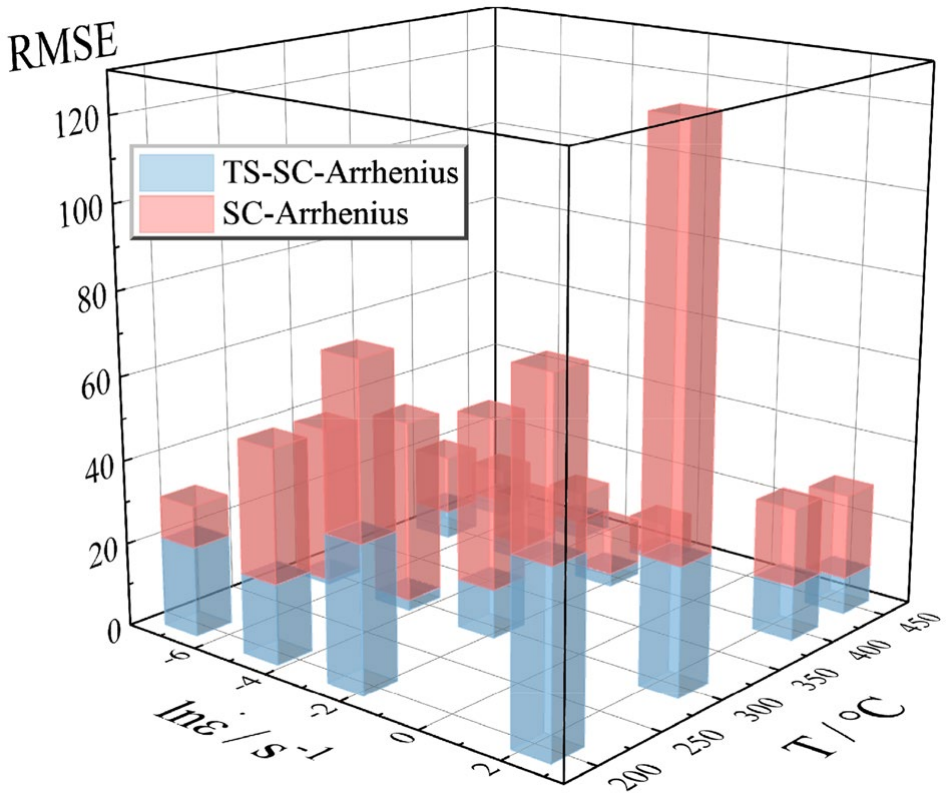
Comparison of RMSE for the SC and TS-SC Arrhenius model (the red columnar is RMSE of SC-Arrhenius and the blue columnar is RMSE of TS-SC-Arrhenius).

AARE may amplify the effect of the error when the evaluation data is smaller. In order to reasonably compare the error distribution between the SC and the TS-SC Arrhenius model, the RMSE are also evaluated for the predicted stress values of the two constitutive models under each deformation condition. From the histogram results of root mean square error (RMSE) (Fig. 11), the accuracy of the TS-SC Arrhenius model is improved under most deformation conditions, especially above the RT. The RMSE of the TS-SC Arrhenius model is smaller, with favorable prediction capability.

### Hot processing map and corresponding deformation behavior

The hot processing map is widely used in the analysis of the hot deformation mechanism of material as well as in the optimization of process parameters^18,25^. According to the DMM proposed by Prasad^34^, the energy consumption of a material during the deformation stage consists of two main components: (1) heat energy *P* generated and released during plastic deformation of the material, and (2) energy consumed *J* by the evolution of the microstructure.

The energy consumption of *P* and *J* can be calculated from the σ, *ε* and $\dot{\varepsilon }$ as expressed in Eqs. (25), (26), and the proportion between them is determined by the strain-rate sensitivity factor *m* at a given stress (Eq. (27)).

25 $$ P = {\upsigma  \dot{\varepsilon } = }{\text{G}} + J = \int_{0}^{{{\dot{\upvarepsilon}} }} {\upsigma d{\dot{\upvarepsilon}} } + \frac{m}{m + 1}{\upsigma  \dot{\varepsilon }} $$

26 $$ m = \left. {\frac{{\partial \left( {\ln\upsigma } \right)}}{{\partial \left( {\ln {\dot{\upvarepsilon}} } \right)}}} \right|_{{T,\upvarepsilon }} $$

27 $$\upeta = \frac{2m}{{m + 1}} $$

**Figure 12:**
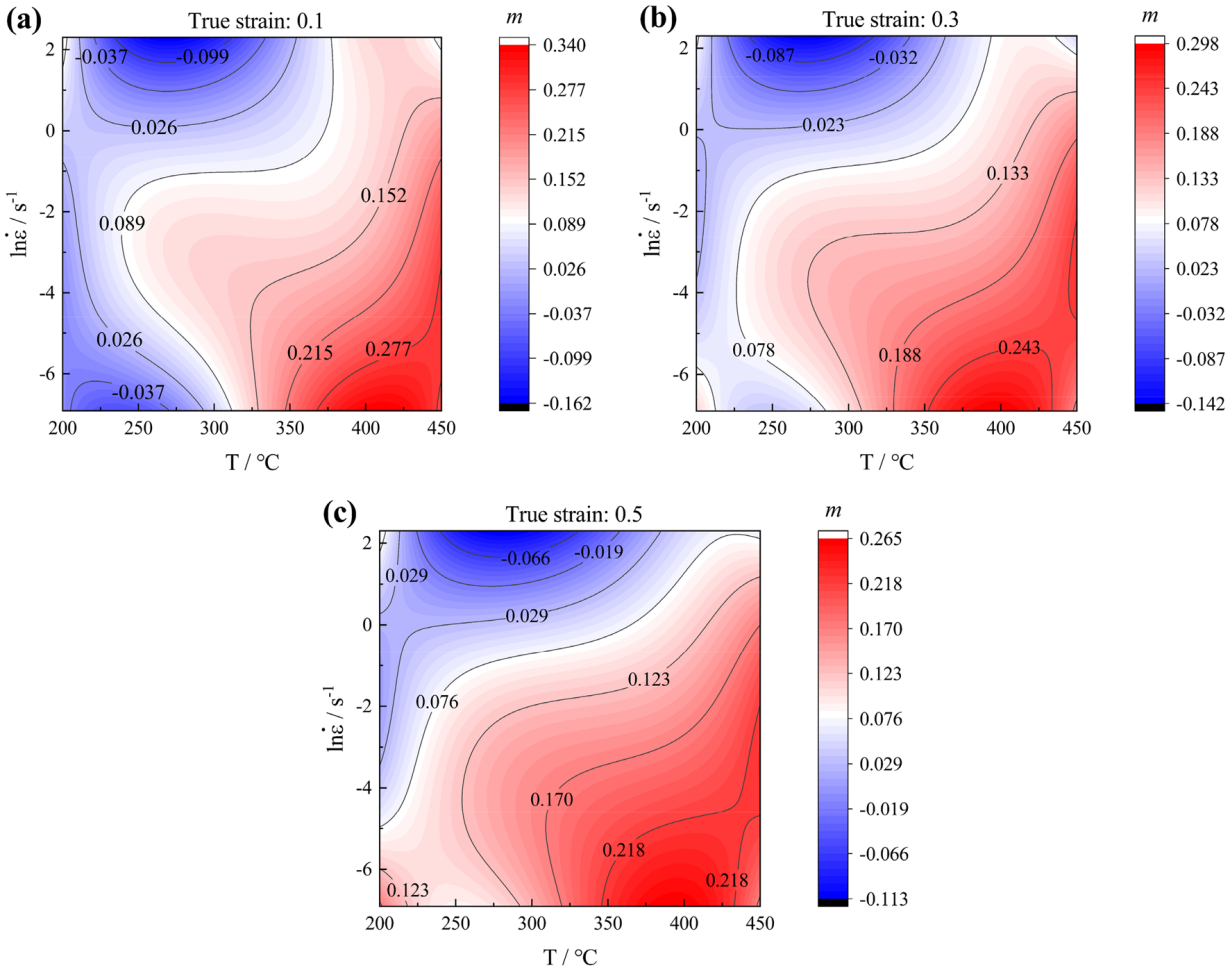
The value of *m* at different true strain: (**a**) 0.1; (**b**) 0.3; (**c**) 0.5.

Murty et al.^35^ argued that the strain rate sensitivity factor *m* is temperature- and strain rate-dependent and that the power dissipation factor estimated by Eq. (27) is inaccurate. Thus, Murty et al.^35^ developed an improved instability criterion in which Eq. (28) is used to calculate the power dissipation factor and Eq. (29) is used to establish the instability criterion. According the Eq. (26), the values of *m* was calculated by gradient function in MATLAB. Figure 12a–c shows the value of m for various deformation conditions at strain of 0.1, 0.3 and 0.5 after interpolation. At different strain, the value of *m* is distributed in approximately the similar way. The locations of the *m* peak values under different strain are in the regions of higher temperature, lower strain rate. The locations of the *m* valley values under different strain are in the regions of lower temperature, lower strain rate or higher strain rate.

**Figure 13:**
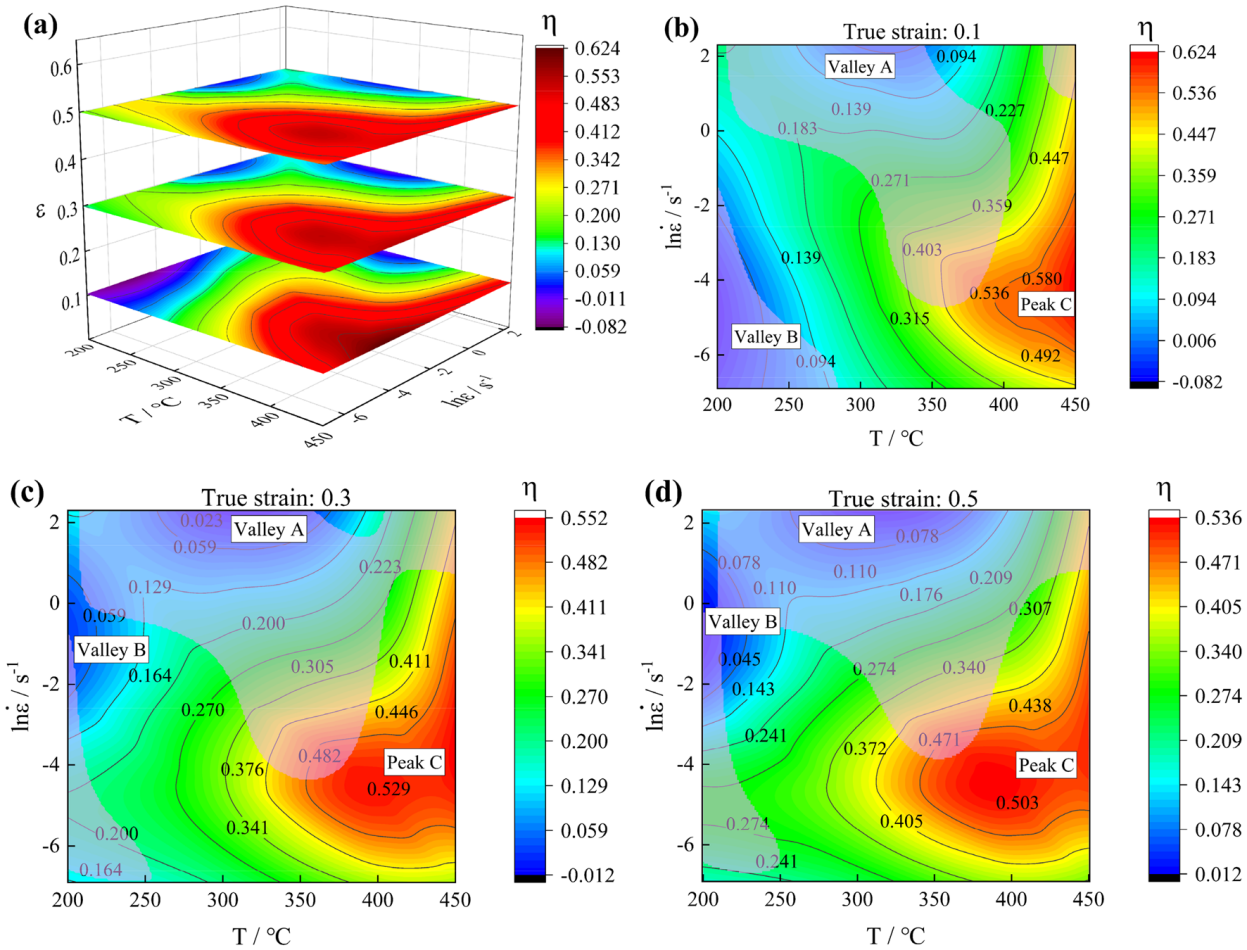
Hot processing map: (**a**) Three-dimensional power dissipation map; (**b**) True strain is 0.1; (**c**) True strain is 0.3; (**d**) True strain is 0.5.

Based on Murty improved DMM, three-dimensional power dissipation coefficient maps and hot processing map of Al–Zn–Mg–Cu alloy are established in this study, as shown in Fig. 13.

28 $$\upeta = \frac{2J}{P} = \frac{{2\left( {P - G} \right)}}{P} = 2{ \times }\left( {1 - \frac{{\left( {\frac{{{\upsigma  \dot{\varepsilon }}}}{m + 1}} \right)_{{{\dot{\upvarepsilon}} =\upvarepsilon _{\min } }} + \int_{{{\dot{\upvarepsilon}} _{\min } }}^{{{\dot{\upvarepsilon}} }} {\upsigma d{\dot{\upvarepsilon}} } }}{{{\upsigma  \dot{\varepsilon }}}}} \right) $$

29 $$\upxi = \frac{2m}{\upeta } - 1 < 0 \, $$

In the hot processing map (Fig. 13), the contour lines represent the power dissipation factor η, and the transparent solid-colored areas represent the unstable regions. The hot processing map also show the power dissipation factor peaks and valleys at each strain. The peak power dissipation factor region of Al–Zn–Mg–Cu alloy shows an increasing trend with increasing strain in the three-dimensional power dissipation factor map, while the value of the peak power dissipation factor steadily declines. Under selected true strains, the tendency of η for Al–Zn–Mg–Cu alloy is to grow gradually with increasing temperature and drop gradually with increasing strain rate.

According to Fig. 13a–d, the high-power zone of Al–Zn–Mg–Cu alloy is located in a deformation condition characterized by high temperature and low strain rate. Conversely, the low power zones are found in regions of low deformation temperature, while the instability zones are primarily concentrated in areas of low temperature and high strain rate. These characteristic zones may be related to the microstructural evolution of Al–Zn–Mg–Cu alloy during hot deformation.

**Figure 14:**
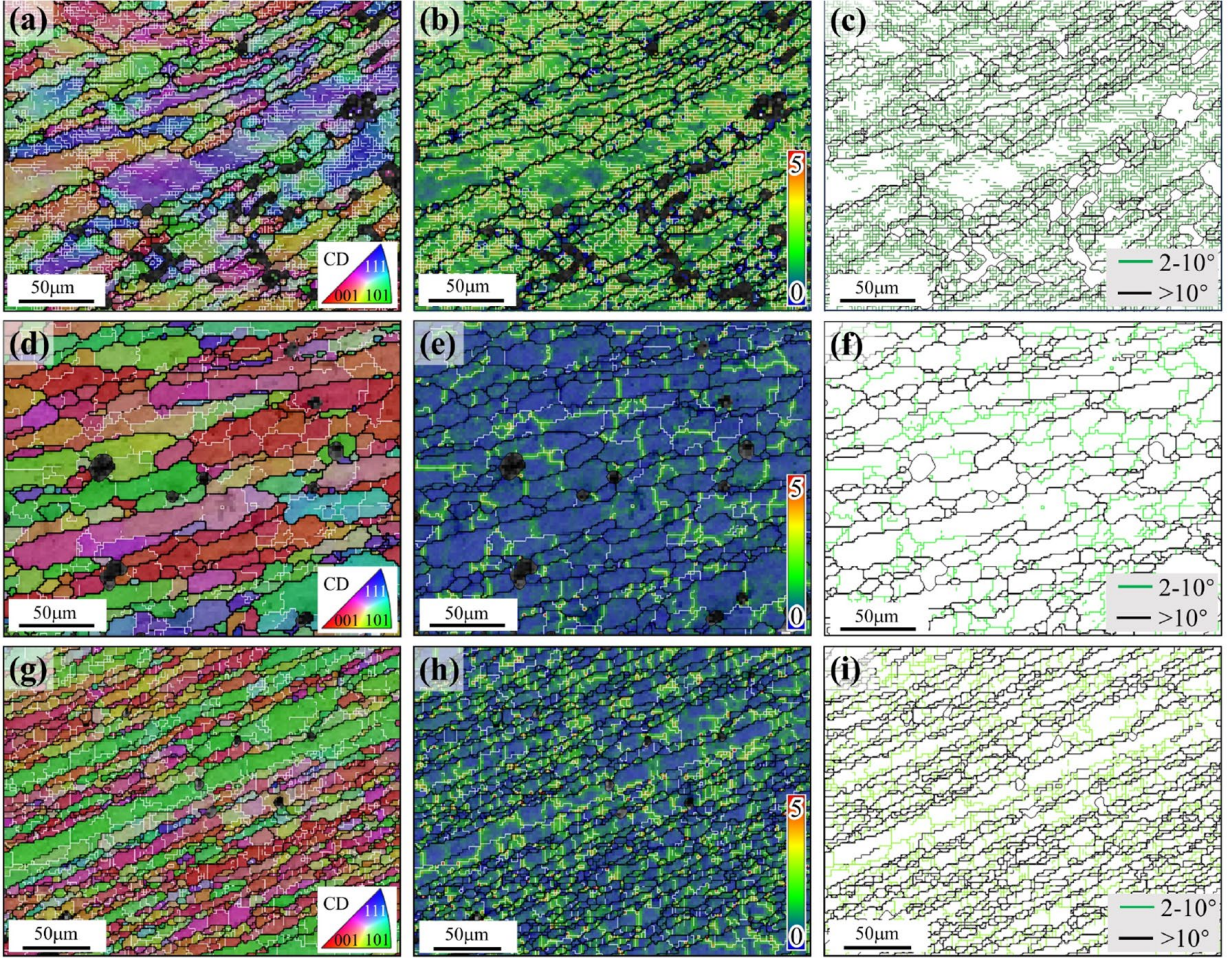
EBSD micrographs of Al–Zn–Mg–Cu alloy: (**a**) IPF map of 300 °C &10 s^−1^; (**b**) KAM map of 300 °C &10 s^−1^; (**c**) GB map of 300 °C &10 s^−1^; (**d**) IPF map of 450 °C &0.01 s^−1^; (**e**) KAM map of 450 °C &0.01 s^−1^; (**f**) GB map of 450 °C &0.01 s^−1^; (**g**) IPF map of 400 °C &0.01 s^−1^; (**h**) KAM map of 400 °C &0.01 s^−1^; (**i**) GB map of 400 °C &0.01 s^−1^.

Samples at 450/400 °C & 0.01 s^−1^ and 300 °C &10 s^−1^ deformation conditions were microstructurally characterized and analyzed in order to understand the microstructural characteristics of the high power and instability zones of Al–Zn–Mg–Cu alloy after hot deformation. The inverse pole figure (IPF), Kernel Average Misorientation (KAM) and grain boundary (GB) maps are plotted in Fig. 14 for the core of the hot compression specimen under deformation conditions of 300 °C &10 s^−1^ and 450/400 °C &0.01 s^−1^, respectively. In the IPF and GB maps, black boundaries represent HAGBs, and white/green boundaries represent LAGBs. And the KAM map is used to describe the level of plastic deformation^36,37^.

Figure 14a–c shows the microstructure under the deformation conditions of 300 °C &10 s^−1^. From the IPF and GB maps, it can be seen that the grain structure consisted of elongated grains and fragmentation grains, with numerous LAGBs inside the elongated grains, which corresponds to the characteristics of the DRV of aluminum alloys. From the KAM map, the higher KAM values at the grain boundaries and interiors indicate that substantial dislocations have accumulated within grains^37^. Combined with the hot processing map, this deformation condition is located in the instability region, which implies that the high strain storage energy increases the possibility of forming defects and that DRX is difficult to occur at low temperatures and high strain rates.

Figure 14d–f shows the microstructure under the deformation conditions of 450 °C &0.01 s^−1^. From the IPF and GB maps, the grain structure consists of elongated deformed grains as well as equiaxed recrystallized grains, which indicates that DRX has occurred under this deformation condition. From the KAM map, the lower KAM values at the grain boundaries and interiors imply that the consumption of dislocations is the combined effect of small-scale DRX and enhanced-DRV.

From the IPF, GB and KAM maps (Fig. 14g–i), it can be seen that the DRX has also occurred under 400 °C &0.01 s^−1^. However, the nucleation rate of DRX under 400 °C &0.01 s^−1^ is faster and the growth rate is slower compared to 450 °C & 0.01 s^−1^. It is because as the deformation temperature rises (400 °C to 450 °C), the atomic diffusion, motion of dislocations, and migration of sub-grain and grain boundaries are enhanced. By this means, dislocation annihilation and rearrangement are accelerated. Therefore, the level of DRV increases such that larger sub-grains with neatly organized boundaries develop. Similarly, with increasing temperature, DRX grains grow faster because of faster grain boundary migration.

**Figure 15:**
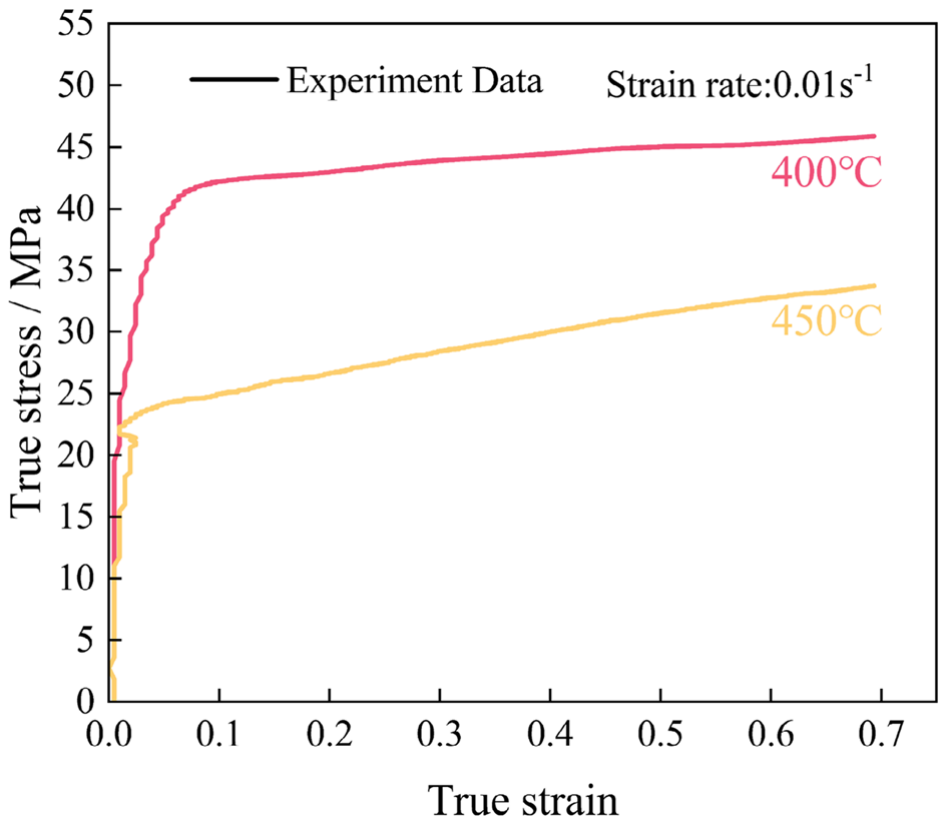
The true stress-true strain curve of 400 °C &0.01 s^−1^ and 450 °C &0.01 s^−1^.

From the point of view of combining macro-curves with microstructures, the macroscopic stress–strain curve of 400 °C &0.01 s^−1^ and 450 °C &0.01 s^−1^ (Fig. 15) displaying no significant curvature change contradicts the microstructure, this is due to the fact that small-scale DRX does not induce significant curvature changes, and similar phenomena are reflected in the macroscopic curves and microscopic features in the relevant literature^9,38^. In addition, the large nucleation rate in the deformation condition of 400 °C&0.01 s^−1^ resulted in the formation of more DRX grains and the refinement of the average grains, leading to a smoother macroscopic stress–strain curve compared with that in the 450 °C&0.01 s^−1^ deformation condition.

### High-temperature softening mechanisms

For deeper understanding of softening mechanisms at high temperatures and low strain rates, DRV and DRX mechanism were studied in this paper, respectively. can be determined by comparing thermal activation energy and apparent activation volume. However, considering that the average slope can cause large errors in the activation energy calculation under the wide deformation conditions, only the apparent activation volumes were compared to preliminarily determined the DRV mechanisms in this paper. The apparent activation volume can be calculated by Eq. (30)^39^. After comparing the apparent activation volumes of the Al–Zn–Mg–Cu alloys studied in this paper (Table 4) with those of pure aluminum (Table 5), it was preliminarily determined that the main DRV mechanism is screw dislocation cross-slip. This is consistent with the DRV mechanism under high temperature deformation of Al–Zn–Mg–Cu alloys in the related literature^40^.

30 $$ V = kT\left( {\frac{{\partial \ln {\dot{\upvarepsilon}} }}{{\partial\upsigma }}} \right)_{T} $$

where *V* is apparent activation volume, *k* is Boltzmann constant, T is deformation temperature, ${\dot{\upvarepsilon}}$ is strain rate and σ is true stress.

**Table 4:** Thermal activation energy and apparent activation volume of Al–Zn–Mg–Cu alloy.

Deformation temperature/°C	Apparent activation volume/nm^3^
200	35.660
300	49.202
400	55.472
450	56.615

**Table 5:** Thermal activation energy and apparent activation volume of pure aluminum^39^.

Dynamic recovery mechanism	Apparent activation volume/nm^3^
Dislocation climb	1
Non-conservative motion of jogs	100–1000
Unzipping attractive junctions	90–600
Cross-slip	10–100

**Figure 16:**
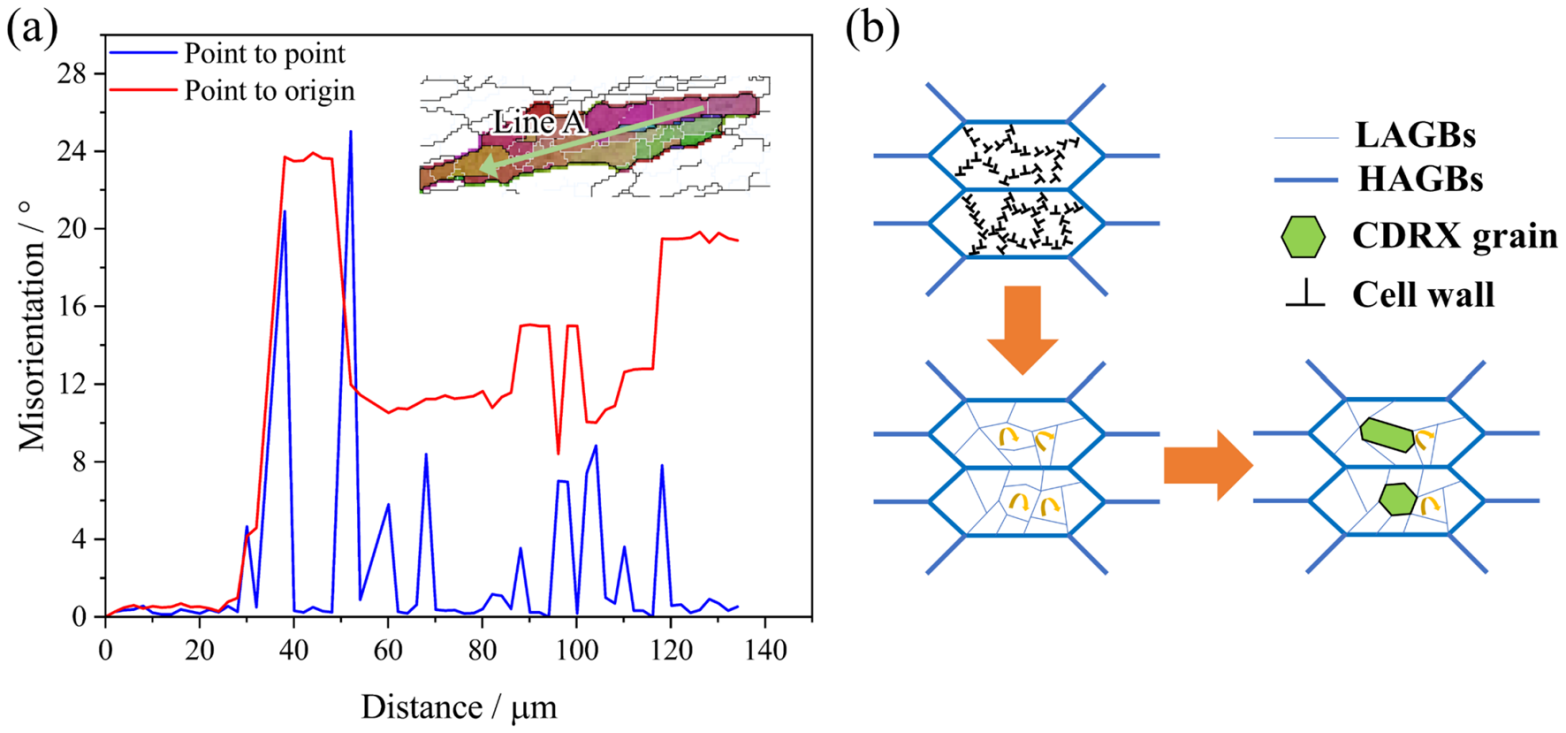
CDRX mechanism: (**a**) Variation of orientation difference; (**b**) Schematic diagram of mechanisms of CDRX.

In order to analyze the DRX mechanism under deformation condition of 450 °C &0.01 s^−1^ in detail, some grains in Fig. 14d are selected for analyzing the intragranular misorientation. Figure 16a gives the distribution of intragranular orientation difference along the green arrow of selected grains. And the blue line and red line in Fig. 16a represent the cumulative orientation difference and the adjacent orientation difference, respectively. When the deformation condition is 450 °C &0.01 s^−1^, the cumulative orientation difference reaching 24° in the selected grain. The curve of cumulative orientation difference is stepwise and has multiple platforms, indicating the presence of some different orientation blocks. The single-step platform is relatively stable, which is consistent with the characteristics of CDRX to produce new grain^5,38^. Figure 16b summarized the CDRX mechanisms of the Al–Zn–Mg–Cu alloy. In the early stage of hot deformation, the dislocations inside the grains are entangled to form dislocation cells. With further deformation, the orientation difference between dislocation cells increases to form LAGBs. As deformation increases, sub-grains continue to absorb dislocations. The misorientation angle is thus incremented and transfers to the HAGBs, resulting in the formation of CDRX grains.

### Strain-induced precipitation and the coupling relationship between precipitates and dislocations

Al–Zn–Mg–Cu alloys represent precipitation-hardened materials, exhibiting multi-scale secondary phase precipitations under diverse thermal and strain conditions. Deformation introduces defects, particularly dislocations, enhancing atomic diffusion and clustering, resulting in pronounced secondary phase precipitation. Additionally, the secondary phase's ability to pin dislocations affects the alloy's deformation behavior. Thus, investigating the interplay between secondary phases, crystal defects, and evolving substructures during hot compression is crucial.

Hot compressive deformation at 300 °C and a strain rate of 0.1 s^−1^ falls within the medium power zone of the hot processing map (Fig. 13). However, its experimental curve (Fig. 3c) resembles the trend of the DRX curve. Thus, this study examines the 300 °C and 0.1 s^−1^ deformation behavior more deeply. Additional experiments were conducted on samples subjected to this condition, with maximum deformation reaching up to 80%, for a comprehensive microscopic analysis of the mechanism.

**Figure 17:**
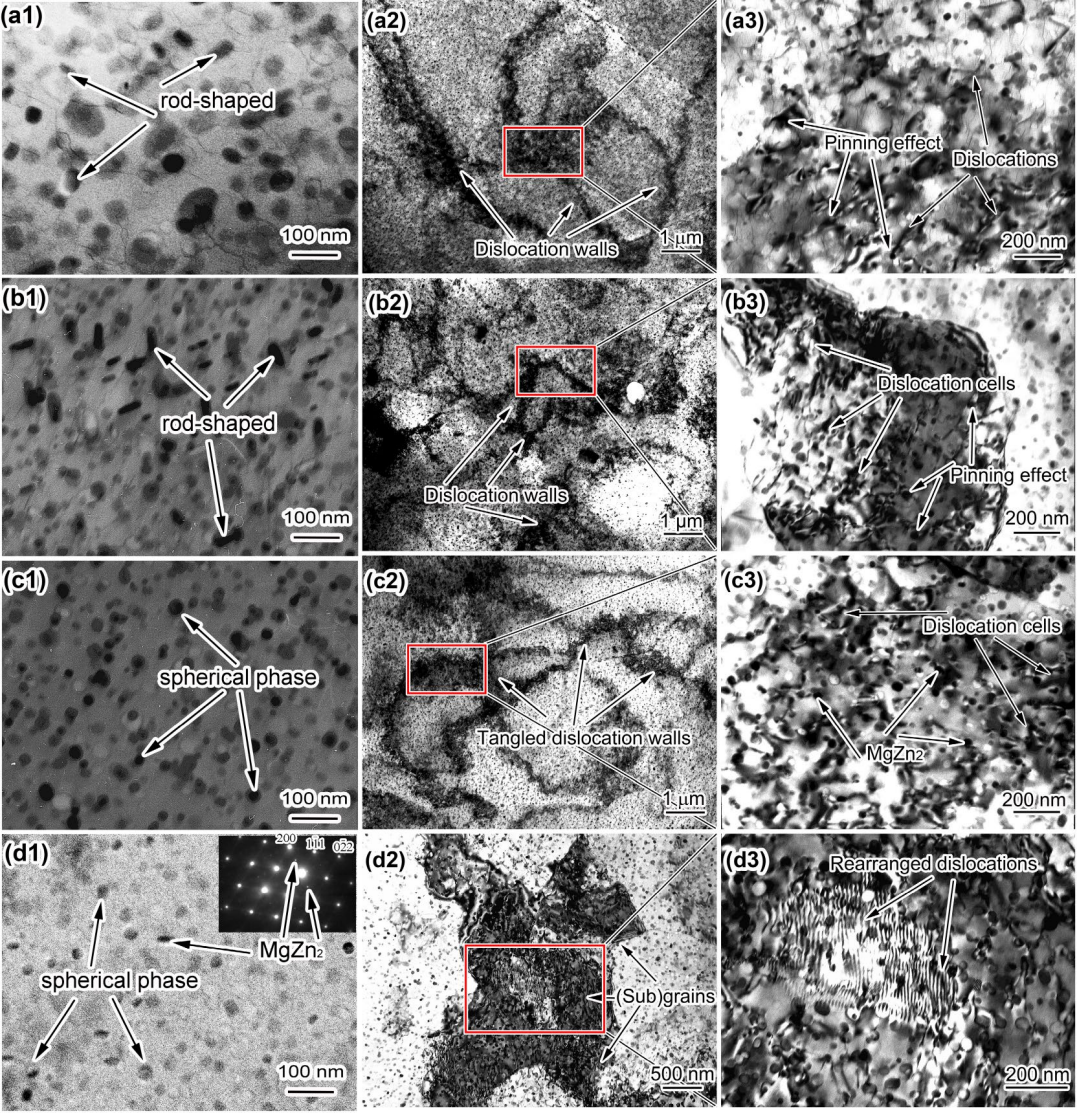
Precipitates and substructure morphology of samples at 300 °C &0.1 s^−1^: (**a1**–**a3**) 20% deformation, where a3 is a locally enlarged image of a2 (the same applies below); (**b1**–**b3**) 40% deformation; (**c1**–**c3**) 60% deformation; (**d1**–**d3**) 80% deformation (bright field image along < 110 > _Al_ crystal plane), where the inset in d1 shows SAED image of precipitates along < 110 > _Al_ crystal plane.

**Figure 18:**
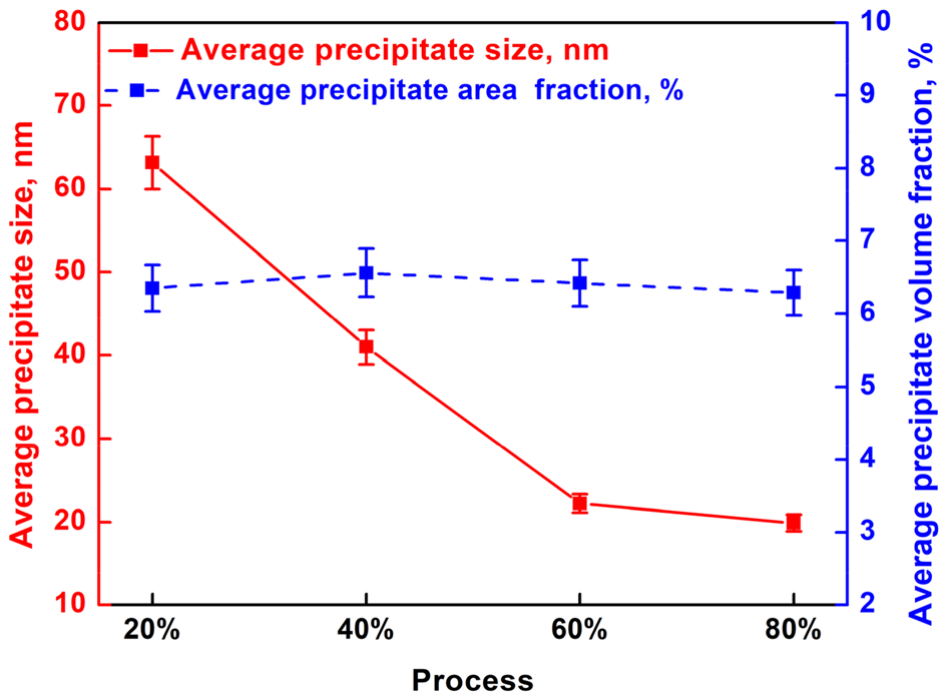
Statistical diagram of precipitates size and area fraction after hot compression with different deformation amounts.

TEM and the selected area electron diffraction (SAED) patterns along the < 110 > _Al_ crystal plane were provided in Fig. 17 to illustrate the evolution of precipitates and substructures (dislocations) following deformation through different processing techniques. Concomitant with plastic deformation, there's a significant surge in the number of precipitates. And these precipitates emerge as finer and more dispersed entities, with an increasing propensity for spheroidization (as depicted in the morphological characterization in Fig. 17a1–d1 and the statistical analysis regarding precipitates in Fig. 18). The area fraction of the precipitates remains largely constant with increasing strain, but a distinct size diminution is observed (Fig. 18). The precipitates change shape, with fewer rod-shaped entities and more spherical entities (refer to Fig. 17a1–d1). Such observations show the influence of strain-induced precipitation. According to the Zener pinning force formula^41^:

31 $$ P_{z} = \frac{{3Fv{ \cdot }r_{b} }}{d} $$

where, *P*_*Z*_ is the zener pinning force, *F*_*v*_ is the volume fraction of precipitated phase, *r*_*b*_ is a constant, and *d* is the average grain diameter. It can be known that the smaller the size of the precipitate and the larger the volume fraction, the stronger its pinning force, which helps to pin sub-grain boundaries in the later stage.

As the amount of deformation increases, the density of dislocations does not continue to rise consistently. Instead, it gradually increases and peaks at a deformation of 60%. Subsequently, as deformation continues, the DRV phenomenon intensifies, leading to a decrease in dislocation density. At a thermal deformation of 20%, dislocation walls begin to form (Fig. 17a2,a3). When the deformation reaches 40%, the dislocation walls strengthen further, and dislocation cells start to appear (Fig. 17b2,b3). After a deformation of 60%, dislocation tangles are most severe, with a high density of uniformly sized dislocation cells (Fig. 17c2,c3). However, when the deformation amount reaches 80%, DRV becomes particularly intense, and sub-grains (substructures) begin to form (Fig. 17d2,d3), further contributing to the decrease in dislocation density.

The relationship between dislocation density ρ and strain ε is as follows^42^:

32 $$\upvarepsilon =\uprho bL $$

where *b* is the Burgers vector associated with the dislocation, *L* is the mean displacement distance of the dislocation, ρ is the dislocation density, and ε is the true strain. During high-temperature deformation of aluminum alloys, increasing strain leads to enhanced dislocation proliferation, elevating dislocation density and stored energy. However, further strain induces significant migration of dislocations and grain boundaries, increasing the average distance of dislocation movement (*L*). This intensifies DRV, where dislocations are rearranged and annihilated, ultimately reducing dislocation density. Thus, while an initial increase in density occurs, DRV eventually reaches an equilibrium state, preventing continuous increase.

**Figure 19:**
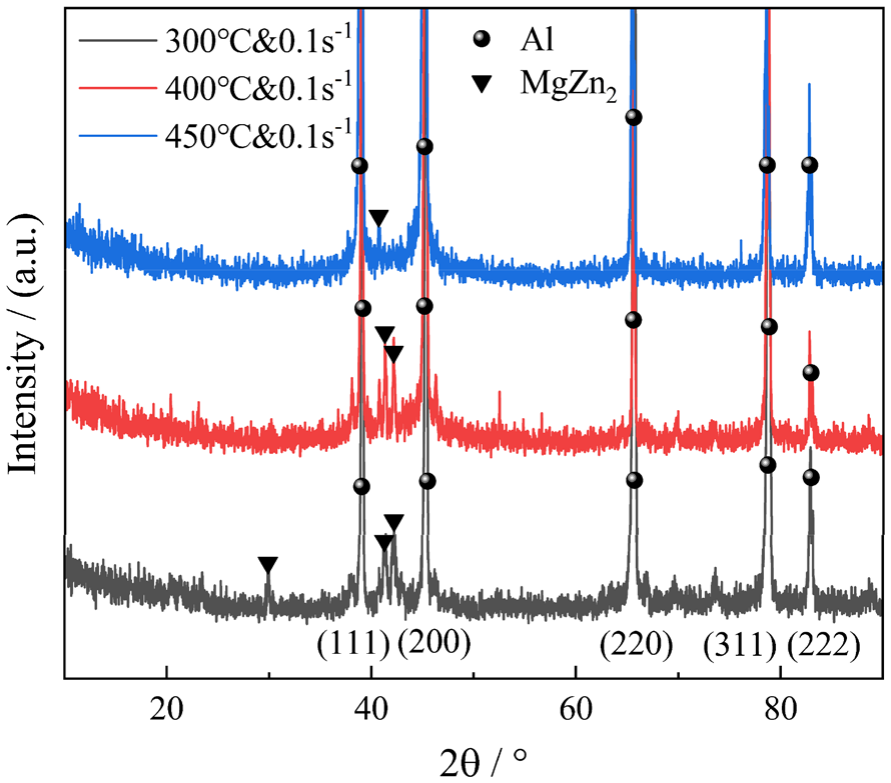
XRD patterns of compressed alloys with various temperatures.

Figure 19 presents the XRD patterns of the alloy subjected to compression deformation at various temperatures. In the analysis of XRD spectra of 7055 aluminum alloy from references, it was observed that the prominent diffraction peaks of the MgZn_2_ phase are typically found at approximately 32.0° and 41.6° 2θ positions^43–45^. The XRD patterns from this experiment were matched against the peak positions reported in the references to confirm the presence of the MgZn_2_ phase in the 7055 aluminum alloy studied in this paper. Furthermore, the intensity of the diffraction peaks was examined qualitatively to determine its content corroborating the selected area electron diffraction results from the TEM analysis in Fig. 17. In the XRD patterns, the intensity of the secondary phase diffraction peaks directly correlates with its content in the alloy. Consequently, an initial increase followed by a decrease in the precipitate phase content is observed with rising deformation temperatures. The solubility of solute atoms Mg and Zn in the aluminum matrix decreases with decreasing temperature. However, the precipitation of the secondary phase is governed by atomic diffusion. At lower deformation temperatures, despite a higher tendency for precipitation, the content of the secondary phase remains low due to the reduced atomic diffusion rate. Conversely, near the solid solution temperature of 450 °C, the increased atomic diffusion rate is offset by the higher solubility of solute atoms, reducing the precipitation tendency. Thus, the content of the secondary phase increases initially and then decreases with increasing temperature.

### Modeling analysis for strain-induced precipitation and DRV/DRX mechanism

**Figure 20:**
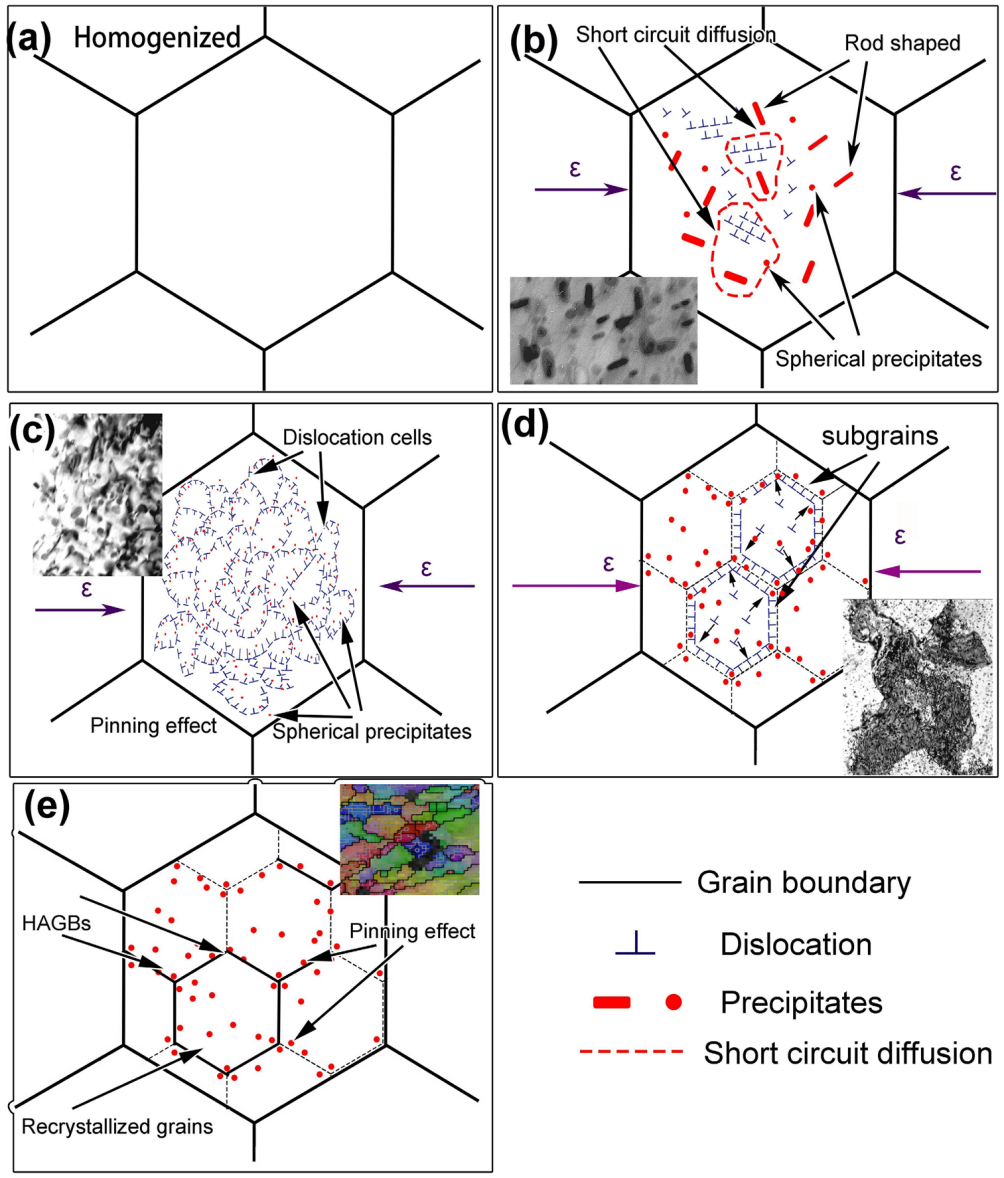
Strain-induced precipitation and DRV/DRX mechanism diagrams: (**a**) Aluminum alloy after homogenization treatment; (**b**) Deformation generates dislocations, enabling short-circuit diffusion for precipitates; (**c**) Further deformation refines and spheroidizes precipitates, initiating DRV and dislocation cell formation; (**d**) As recovery progresses, cell interiors purify, and dislocations amass at walls, generating sub-grains; (**e**) precipitates pin boundaries, increasing orientation differences transition LAGBs to HAGBs, producing fine recrystallized grains.

Thermodynamically, structures with elevated Gibbs free energy transition to states with reduced Gibbs free energy, inducing nucleation of new phases. Following homogenization treatment (Fig. 20a), the Al–Zn–Mg–Cu alloy underwent hot compression, introducing a significant number of defects, such as dislocations, as seen in Figs. 17a2 and 20b. These defects act as efficient diffusion conduits for solute atoms, accelerating their movement (Fig. 20b). Concurrently, these imperfections alter the inherent chemical potential within the grains, reshaping diffusion trajectories and segregation locales for Mg and Zn atoms (Fig. 20b,c). Defects vanish or are disrupted upon nucleation of new phases, releasing energy and diminishing the system's total Gibbs free energy (ΔG), stabilizing microstructures. Heterogeneous nucleation increases sites available for precipitate formation and influences interfacial energy (Δ*G*_*b*_). Among various morphologies, spherical precipitates exhibit the lowest Gibbs free energy, leading to a more stabilized system. The variation in Gibbs free energy during dynamic precipitation can be articulated using the Eq. (33)^46,47^.

33 $$ \Delta G^{\prime} = - V\Delta G_{V} + V\Delta G_{\varepsilon } + A\gamma - V\Delta G_{D} $$

where Δ*G*_*D*_ is the decrease of Gibbs free energy per unit volume after nucleation at defects. The critical nucleation radius R* can be expressed as Refs.^46,47^:

34 $$ R^{*} = \frac{{2\upgamma }}{{\Delta G_{V} + \Delta G_{\varepsilon } + \Delta G_{D} }} $$

During hot deformation, aluminum alloys introduce defects like vacancies and dislocations into their crystal structure. Although microscopic, these defects significantly impact solute atom migration. With lower migration energy barriers compared to perfect crystal lattices, defects act as shortcuts for rapid solute atom diffusion, accelerating nucleation and growth of the second phase. Nucleation involves new phases emerging from supersaturated solid solutions.

Vacancies and dislocations not only facilitate solute atom migration but also provide additional nucleation sites, reducing critical nucleation radius and promoting increased nucleation numbers. This leads to finer precipitate size under deformation, as the increased distribution of nucleation sites limits growth space for each phase, resulting in smaller precipitate sizes. From Eqs. (33), (34), as deformation and Δ*G*_*D*_ increases, enlarging the denominator (Δ*G*_*V*_ + Δ*G*_*ε*_ + Δ*G*_*D*_). This reduces the critical nucleation radius R* while augmenting the precipitation driving force Δ*G’*, thereby boosting the nucleation rate and quantity. As a consequence, precipitate size diminishes, as shown in Figs. 17a1–d1 and 20c.

As deformation increases, interfacial energy per unit volume (γ) rises, surpassing the effect of strain energy (Δ*G*_*ε*_) on nucleation. Spherical particles exhibit minimal interfacial energy, driving MgZn_2_ precipitates to spheroidize and optimize Gibbs free energy. Thus, deformation-induced precipitates predominantly adopt a spherical morphology, as seen in Fig. 17. Solute atom behavior, particularly desolvation and secondary phase particle precipitation, primarily results from diffusion and solute segregation processes. During hot deformation, defects like sub-grain boundaries, dislocations, and vacancies emerge, which can provide more nucleation points for the second-phase precipitation, and can also become a rapid diffusion channel of atoms in the solid solution to accelerate atomic diffusion, similar phenomenon had been obtained by Starke and Troeger^48^. Alloying elements like Mg and Zn migrate towards these defects, leading to atom clustering and enrichment. This alters atom diffusion directions and shifts preferred segregation areas. Solute atom migration from rod-shaped precipitates to these regions illustrates a distinct re-dissolution and re-precipitation process, highlighting the intricate relationship between microstructural shifts and material deformation.

The interplay between secondary phase and dislocations influences the alloy's DRV and DRX. Refined precipitates pin dislocations, forming relatively dislocation-free cells with concentrated arrays at their boundaries (Fig. 20c). Continued deformation increases dislocation density at these boundaries, thickening and structuring them. Intersecting boundaries or aligned dislocations within transform into LAGBs, transitioning initial dislocation cells into sub-grains (Fig. 20d). As deformation progresses, precipitates pin sub-grain boundaries, amplifying orientation disparities and transforming LAGBs into HAGBs, initiating DRX (Fig. 20e). Softening induced by DRV and DRX counteracts contributions from precipitation strengthening and work hardening, stabilizing material flow. In the stable flow state, an approximate equilibrium between strengthening and softening mechanisms maintains stress levels without significant fluctuations (Fig. 3c).

## Conclusion

Uniaxial compression tests on Al–Zn–Mg–Cu alloy samples were conducted on a Gleeble-1500 thermal simulator at temperatures ranging from 200 °C to 450 °C and strain rates from 0.001 s^−1^ to 10 s^−1^. True strain–stress curves were double-corrected to account for temperature fluctuations and friction during hot deformation. Key conclusions are as follows:

1. The SC Arrhenius model was established with material parameters α, *n*, *Q*, and ln*A* as polynomial fitting functions about strain ε.
2. A TS-SC Arrhenius constitutive model was developed. Assessment based on R-value, AARE-value, and RMSE-value showed higher accuracy for the TS-SC Arrhenius model over a wider temperature range compared to the SC Arrhenius model.
3. Hot processing map and EBSD analysis revealed microstructure evolution dominated by DRV at low-temperature and high strain rates as well as small-scale DRX and DRV at high-temperature and low strain rates.
4. Hot deformation in the solid-solution state generates crystal defects, accelerating solute atom diffusion and leading to strain-induced precipitation. Strain energy governs rod-shaped precipitates under over-aging or suboptimal deformation, while interfacial energy predominates with further deformation, promoting spheroidal precipitate evolution. This synergistic interaction between precipitates and dislocations facilitates both DRV and small-scale DRX phenomena.

## Data Availability

The datasets used and/or analyzed during the current study available from the corresponding author on reasonable request.
